# A shift between mineral and nonmineral sources of iron and sulfur causes proteome-wide changes in *Methanosarcina barkeri*

**DOI:** 10.1128/spectrum.00418-23

**Published:** 2024-01-05

**Authors:** Hunter Fausset, Rachel L. Spietz, Savannah Cox, Gwendolyn Cooper, Scott Spurzem, Monika Tokmina-Lukaszewska, Jennifer DuBois, Joan B. Broderick, Eric M. Shepard, Eric S. Boyd, Brian Bothner

**Affiliations:** 1 Department of Chemistry and Biochemistry, Montana State University, Bozeman, Montana, USA; 2 Department of Microbiology and Cell Biology, Montana State University, Bozeman, Montana, USA; Politecnico di Torino, Torino, Italy

**Keywords:** proteomics, shotgun proteomics, methanogens, mineral, variable phenotypes, iron sulfur, iron reduction, omics

## Abstract

Iron (Fe) and sulfur (S) are required elements for life, and changes in their availability can limit the ecological distribution and function of microorganisms. In anoxic environments, soluble Fe typically exists as ferrous iron [Fe(II)] and S as sulfide (HS^−^). These species exhibit a strong affinity that ultimately drives the formation of sedimentary pyrite (FeS_2_). Recently, paradigm-shifting studies indicate that Fe and S in FeS_2_ can be made bioavailable by methanogens through a reductive dissolution process. However, the impact of the utilization of FeS_2_, as opposed to canonical Fe and S sources, on the phenotype of cells is not fully understood. Here, shotgun proteomics was utilized to measure changes in the phenotype of *Methanosarcina barkeri* MS grown with FeS_2_, Fe(II)/HS^−^, or Fe(II)/cysteine. Shotgun proteomics tracked 1,019 proteins overall, with 307 observed to change between growth conditions. Functional characterization and pathway analyses revealed these changes to be systemic and largely tangential to Fe/S metabolism. As a final step, the proteomics data were viewed with respect to previously collected transcriptomics data to deepen the analysis. Presented here is evidence that *M. barkeri* adopts distinct phenotypes to exploit specific sources of Fe and S in its environment. This is supported by observed protein abundance changes across broad categories of cellular biology. DNA adjacent metabolism, central carbon metabolism methanogenesis, metal trafficking, quorum sensing, and porphyrin biosynthesis pathways are all features in the phenotypic differentiation. Differences in trace metal availability attributed to complexation with HS^−^, either as a component of the growth medium [Fe(II)/HS^−^] or generated through reduction of FeS_2_, were likely a major factor underpinning these phenotypic differences.

**IMPORTANCE:**

The methanogenic archaeon *Methanosarcina barkeri* holds great potential for industrial bio-mining and energy generation technologies. Much of the biochemistry of this microbe is poorly understood, and its characterization will provide a glimpse into biological processes that evolved close to life’s origin. The discovery of its ability to extract iron and sulfur from bulk, solid-phase minerals shifted a longstanding paradigm that these elements were inaccessible to biological systems. The full elucidation of this process has the potential to help scientists and engineers extract valuable metals from low-grade ore and mine waste generating energy in the form of methane while doing so.

## INTRODUCTION

All organisms require iron (Fe) and sulfur (S) as essential components of vitamins and a wide range of co-enzymes and co-factors, with only one characterized exception (*Lactococcus plantarum*) (1). [Fe-S] clusters, when coordinated by a protein or protein complex, have a variety of roles in biological systems including electron transfer, substrate binding, and catalysis (2, 3). Therefore, it is not surprising that [Fe-S] clusters are widely distributed across the proteomes in all three domains of life, in processes as varied as photosynthesis, respiration, and fermentation (4–6).

Microorganisms typically acquire Fe by direct transmembrane transport of ferrous iron [Fe(II)] or with the aid of extracellular metal chelators known as siderophores (7, 8). The mechanism of Fe uptake largely depends on the form of Fe available to an organism. Ferric [Fe(III)] iron is the most abundant form in oxic conditions and ferrous [Fe(II)] in anoxic environments (8). Fe(III) has low solubility in oxic circumneutral environments; however, secreted siderophores can chelate Fe(III) making it more bioavailable. Like Fe, the availability and form of S are dependent on environmental conditions. In oxic environments, inorganic S largely exists as sulfate (SO_4_^2−^), whereas in anoxic environments, sulfide (HS^−^) tends to predominate. S can be acquired as inorganic (e.g., SO_4_^2−^ and HS^−^) or organic [e.g., cysteine (Cys)] forms (9). Regardless of the form acquired, reduced forms of S are preferred as this is the oxidation state most often found in co-factors and amino acids. Intricate pathways have evolved to reduce and assimilate SO_4_^2−^ as HS^−^ and to liberate S from organic sources for use in [Fe-S] clusters, amino acids, and other biomolecules (9).

Methanogens are anaerobic archaea that generate methane as a product of the metabolism of hydrogen and carbon dioxide, formate, methanol, methylamines, and/or acetate. Due to their unique biology, these organisms require more Fe per cell and encode a higher number of [Fe-S] cluster-binding motifs in proteins when compared with other organisms (10–12). Methanogens are typically grown with Fe(II) as the Fe source and HS^−^ or Cys as a S source. However, Fe(II) and HS^−^ readily react to form soluble [Fe-S] clusters that nucleate and ultimately form low-solubility iron-sulfide minerals, such as pyrite (FeS_2_). As such, higher concentrations of environmental Fe(II) lead to S limitation and higher concentrations of environmental HS^−^ can lead to Fe limitation. How methanogens obtain Fe and S under anoxic conditions that promotes FeS_2_ formation and limitation of either Fe or S is a central question in understanding the ecology and function of these organisms (13).

Recently, the methanogens *Methanococcus voltae*, *Methanococcus maripaludis*, and *Methanosarcina barkeri* were shown to be capable of using FeS_2_ as their sole source of Fe and S through a reductive dissolution process. Reduction of FeS_2_ releases HS^−^ into solution and generates pyrrhotite (Fe_1-x_S) on the mineral surface. Subsequent solubilization of Fe from Fe_1-x_S yields soluble Fe(II) that can react with HS^−^ in solution to from aqueous [Fe-S] clusters (FeS_aq_), the presumed source of Fe and S for cells (13–17). Direct contact between FeS_2_ and cells is required for reduction, implying direct extracellular electron transfer (EET). The mechanism of EET, as well as the process by which cells internalize and process FeS_aq_ clusters, remains unelucidated (16).

The various forms of Fe and S that can be used to cultivate methanogens provide an opportunity to address such gaps in knowledge. For example, comparison of cells grown with excess HS^−^ and limiting Fe(II), compared with growth with FeS_2_, could provide insight into how cells overcome Fe(II) limitation during growth. Likewise, comparison of cells grown with Cys and Fe(II), when compared with growth with FeS_2_ could shed light into how cysteine decomposition products (e.g., alanine) broadly impact carbon utilization pathways (15, 17). Here, we examine *M. barkeri* strain MS cells grown with HS^−^/Fe(II), Fe(II)/Cys, and FeS_2_ as their sole Fe and S sources. A total proteome analysis under the different conditions was completed to identify specific proteins and biological pathways that are important for growth on different sources of Fe and S. These data were then combined with previously published transcriptomics data (16) to identify changes on a multi-omics level.

## RESULTS

### Shotgun proteomics, statistical analysis, and functional categorization

**Fig 1:**
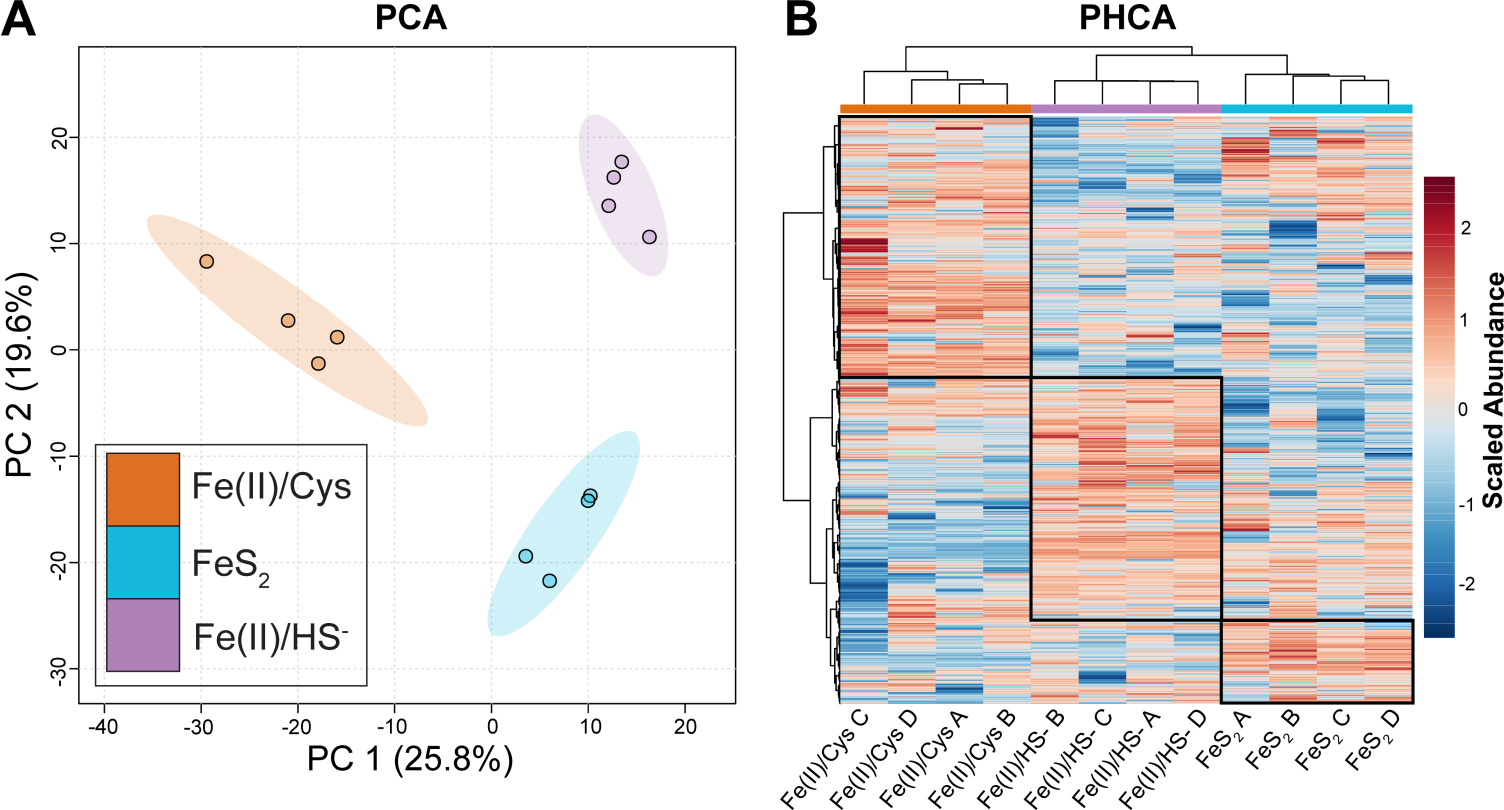
Multivariate statistical analysis of proteomics data. (A) Principal component analysis—PC1 (25.8% of variation) and PC2 (19.6%) coordinates of PCA between cysteine- [Fe(II)/Cys, orange], pyrite- (FeS2, blue), and sulfide- [Fe(II)/HS^−^, purple] grown cells. Colored ovals represent 95% CI. (B) PHCA heatmap. Blocks of coregulated proteins are outlined by black boxes. Dendrogram of samples (x axis) compared with dendrogram of proteins (y axis). A through D indicate biological replicate cultures of each condition. Red and blue indicate abundance above or below average for each protein.

To elucidate proteins involved in Fe assimilation, batch cultures of *M. barkeri* MS were grown on three different forms of Fe and S: Fe(II)/HS^−^ (as FeCl_2_ and Na_2_S), FeS_2_ (as synthetic, framboidal pyrite), and Fe(II)/Cys (as FeCl_2_ and L-cysteine), and harvested according to established protocols (15–17). Cultures (*n* = 4 per condition) were harvested mid-log phase, using methane production and DNA yield as proxies for cell growth (17). A total of 1,019 proteins were used to compare samples. Multivariate analysis based on protein abundance was employed to distinguish proteins with a statistically significant difference between conditions. A total of 307 proteins displayed statistical significance in a one-way analysis of variance (ANOVA) [false discovery rate (FDR)-adjusted *P* < 0.05], representing 30.1% of the data set (Fig. S1), and thus were selected for further analysis and interpretation. Abundance changes ranged from a lower threshold of 2-fold to upwards of 180-fold. Principal component analysis (PCA) was performed to evaluate intra- and intergroup proteomic variabilities. Each growth condition clearly separated when ordinated by PCA, with a roughly equilateral distance between groups in the first two components, together accounting for 45.4% of the total variability of the data set (Fig. 1A). The 95% confidence intervals of each group showed no overlap, indicating that the proteomic changes observed were distinct for each experimental condition. The parallel hierarchical clustering analysis (PHCA) shows the treatment group clustering orthogonal to the pattern of protein abundance clustered by their regulatory patterns (i.e., abundance in one condition relative to the others). Proteins that have consistent abundance changes within a group are clustered together in co-regulated blocks (Fig. 1B).

**Fig 2:**
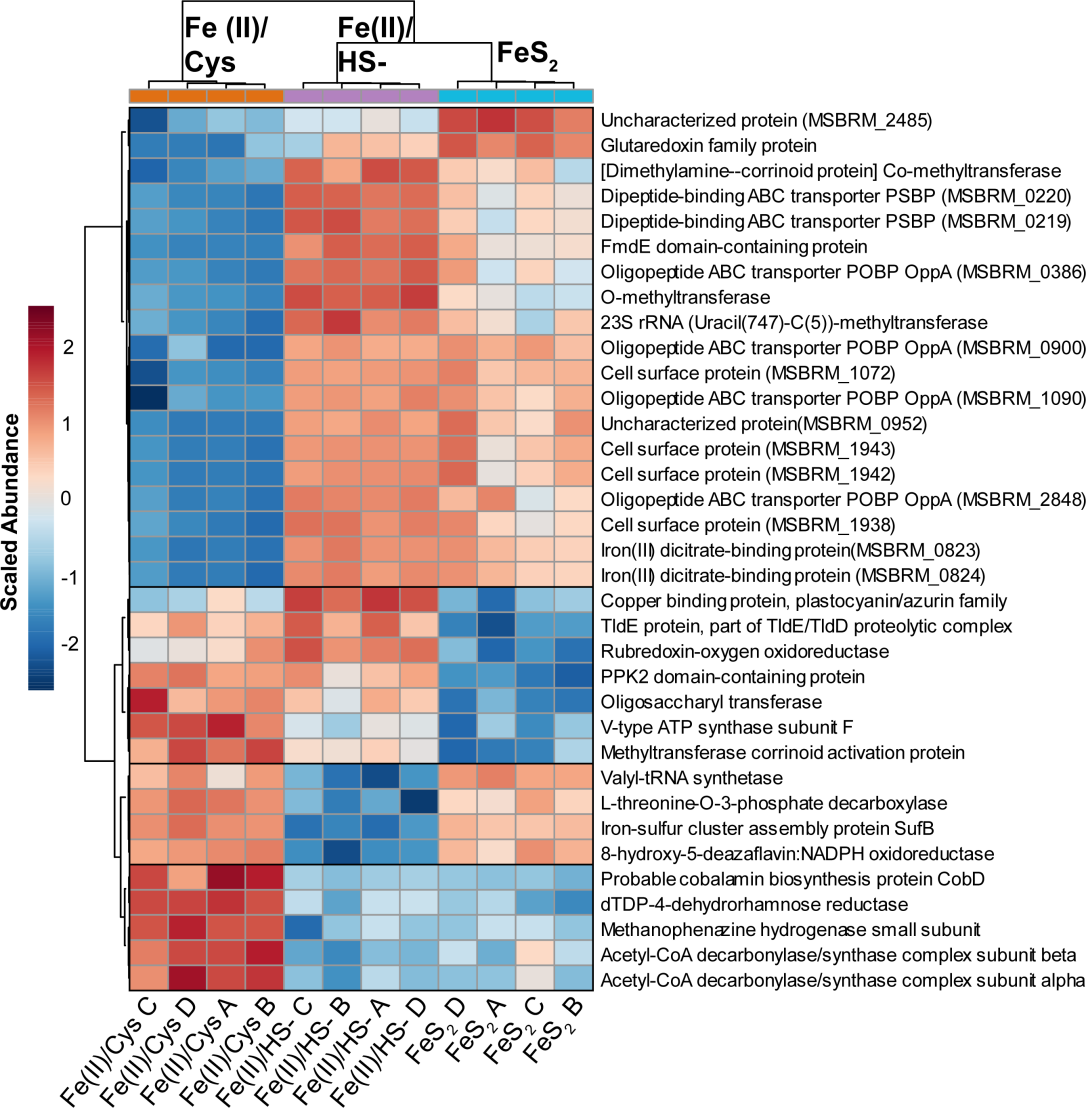
PHCA heatmap of the top 35 proteins (filtered by lowest FDR-adjusted *P*-value, ANOVA). Dendrograms on each axis employ Euclidian distance and a ward clustering algorithm. Y axis dendrogram clusters proteins by abundance, while the x axis clusters samples (biological replicates marked A through D) based on protein abundance patterns. Each block displays the relative, auto-scaled abundance in each sample (red > average, blue < average). Proteins fall into one of five distinct clusters (1–5) (PSBC, periplasmic substrate-binding component; POBP, periplasmic oligopeptide-binding protein).

To help focus on the analysis, a hierarchical clustering analysis (HCA) was repeated using only the 35 top proteins (ranked and filtered by FDR-adjusted ANOVA *P*-value) (Fig. 2). By narrowing our protein list with FDR-corrected *P*-value, rather than abundance, regulatory patterns emerge as clusters on the PHCA heatmap (black boxes in Fig. 2). Five distinct regulatory patterns (clusters) are present in the list. Cluster 1 includes an uncharacterized protein alongside a glutaredoxin family member, both of which are most abundant in the FeS_2_ condition followed by Fe(II)/HS^−^ and are lowest in Fe(II)/Cys condition. The second cluster is the largest and contains di- and oligopeptide-binding ABC transporters, as well as cell surface proteins. Two iron(III) dicitrate-binding proteins that are adjacent in the genome have essentially identical regulation patterns in this cluster. Proteins in cluster 2 are distinctly downregulated in the Fe(II)/Cys condition. Cluster 3 contains a copper binding protein, as well as central carbon and methanogenesis enzymes (V-type ATP synthase and methyltransferase corrinoid activation protein). Clusters 4 and 5 contain secondary metabolism enzymes (L-threonine-O-3-phosphate decarboxylase, iron sulfur cluster assembly protein SufB) and central carbon enzymes (two acetyl-CoA-decarbonylase subunits). Box and whisker plots displaying the regulatory pattern for these 35 proteins are in Fig. S2 and the accompanying protein identifiers in Table S1.

To frame the statistical distance between the groups in terms of biological processes, partial least squares discriminant analysis, a dimensional reduction statistical model, was performed. The top 15 proteins, filtered by VIP score (a metric of statistical weight for differentiating groups), mirror the heatmap in their functional disparity (Fig. S3). The Database for Annotation, Visualization and Integrated Discovery (DAVID) was employed to annotate and assign molecular function Gene Ontology (GO) IDs that correspond to the GO term for each protein (18). Proteins were automatically assigned functions in one or more of the 24 functional categories based on AA sequence similarities. There is clear similarity in the distribution of enzyme function when comparing the proteins that exhibit altered abundance to those that remain unchanged. This is echoed when comparing the analogous transcripts in a previous transcriptomics investigation (Fig. S4) (17). This, combined with the results from the multivariate analysis, suggests that cells undergo global physiological shifts according to growth condition, despite FeS_2_ and Fe(II)/HS^−^ sharing an effective Fe/S source. This prompted a deeper investigation utilizing two-group statistical comparisons to correlate specific Fe and S environment changes with proteomics perturbation.

### Pairwise analysis of conditions

**Fig 3:**
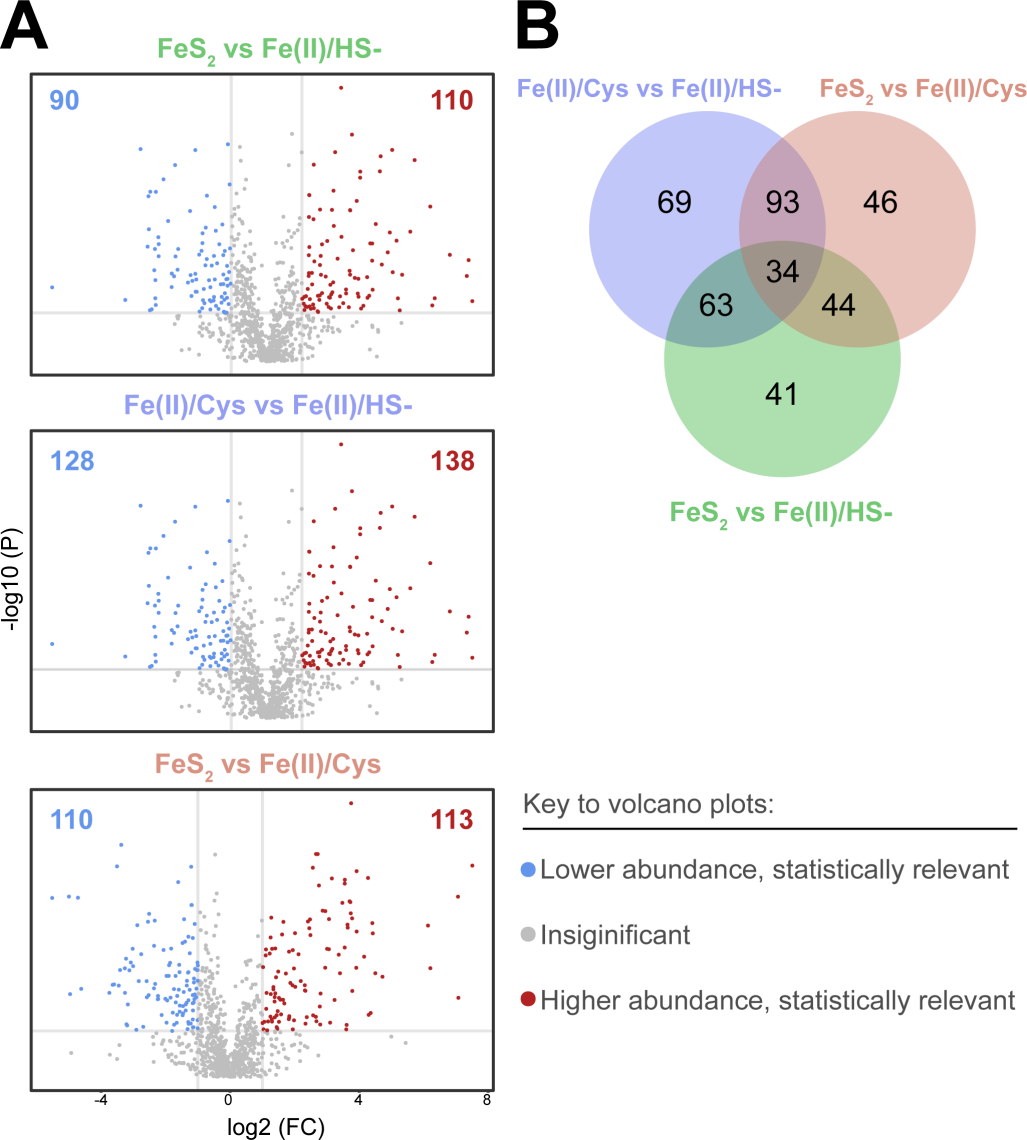
Pairwise comparison of experimental groups. (A) Volcano plots comparing fold change [log2(FC)] with statistical significance [−log10(*P*)] for each comparison. Horizontal line indicates statistical interest threshold (FDR-adjusted *P* < 0.1), vertical line indicates fold change threshold (>twofold higher or lower). Blue/leftward proteins have lower abundance in specified comparison direction (e.g., FeS_2_ vs Fe(II)/HS), and red/rightward proteins have higher abundance [e.g., 90 proteins have significantly lower abundance in FeS_2_ when compared with Fe(II)/HS]. (B) Venn diagram showing which proteins with statistically relevant (FDR-adjusted *P* < 0.1) abundance differences are shared between comparisons. Each comparison is represented by a specific color. Thirty-four proteins were found to be changing by at least twofold in each of the two group comparisons. Sixty-nine proteins were found to be uniquely changing when comparing Fe(II)/Cys to Fe(II)/ HS, 46 when comparing FeS2 to Fe(II)/ Cys, and 41 when comparing FeS2 to Fe(II)/ HS.

To characterize the biological variance between the phenotypes, two-group comparative analyses were performed to link protein patterns with specific cultivation conditions. The statistical analyses were split into three separate two-group analyses (FeS_2_ vs Fe(II)/HS^−^, FeS_2_ vs Fe(II)/Cys, and Fe(II)/Cys vs Fe(II)/HS^−^), wherein *t*-tests were performed on each protein to assess the significance of the difference in mean protein abundances for each growth conditional comparison. The *P*-value threshold was expanded to 0.1 for this and the following functional and biological characterizations to more fully capture shifts in pathway utilization. When assessed in this way, 390 of the 1,019 proteins met the threshold of statistical relevance (FDR-adjusted *P* < 0.1, fold change (FC) > 2) in one or more of the comparisons. Volcano plots of each comparison display a similar degree of proteomic perturbance, with the Fe(II)/Cys and Fe(II)/HS^−^ groups differing the most. All three comparisons show a relatively even distribution of high- and low-abundance proteins which is consistent with unbiased data (Fig. 3A). A Venn diagram of the three pairwise analyses was created to assess which proteins were unique to specific comparisons and which were shared in more than one comparison (Fig. 3B). Thirty-four of the 390 varied in a statistically significant way and shared in all three comparisons.

**Fig 4:**
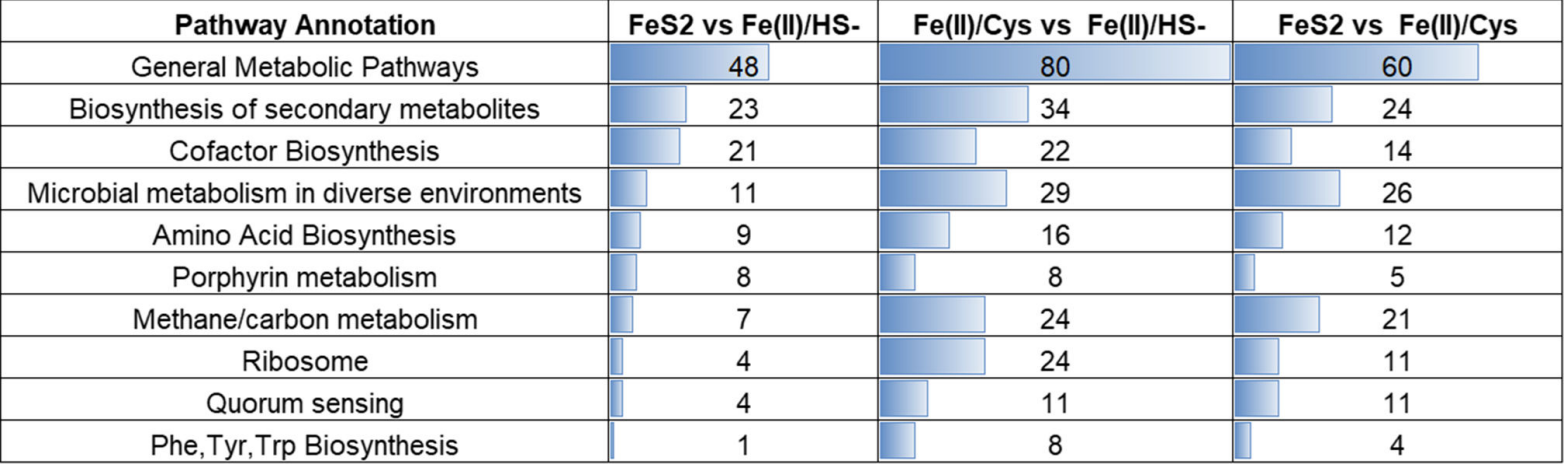
Number of proteins changing in abundance (FDR-adjusted *P* < 0.1) in various KEGG biochemical pathways.

Proteins with different abundances between treatment groups were categorized by the Kyoto Encyclopedia of Genes and Genomes (KEGG) (19) to map the biochemical pathways associated with the observed statistical patterns. The top 10 pathways and the number of proteins of statistical relevance (FDR-adjusted *P* < 0.1, FC > 2) in each analysis are shown in Fig. 4. Notably, Fe(II)/Cys vs Fe(II)/HS^−^ showed a greater degree of abundance differences than comparisons with FeS_2_ in nearly every category. To supplement the pathway analysis, a functional enrichment clustering analysis was performed using DAVID (18) for each two-group statistical comparison (Table S4). Both analyses suggest marked abundance changes in proteins involved in ribosomal biology, methane/carbon metabolism, cell/cell communication, and amino acid-related metabolism, among other functions. The broad scope of proteomic differences observed indicates the involved processes are nuanced and go beyond the aforementioned biological pathways. Because of this, only the most enriched and well-annotated pathways (ranked by overall protein membership and functional categorization) will be used in this investigation to begin to characterize the phenotypes adopted in each growth condition.

### Ribosomal tuning

**Fig 5:**
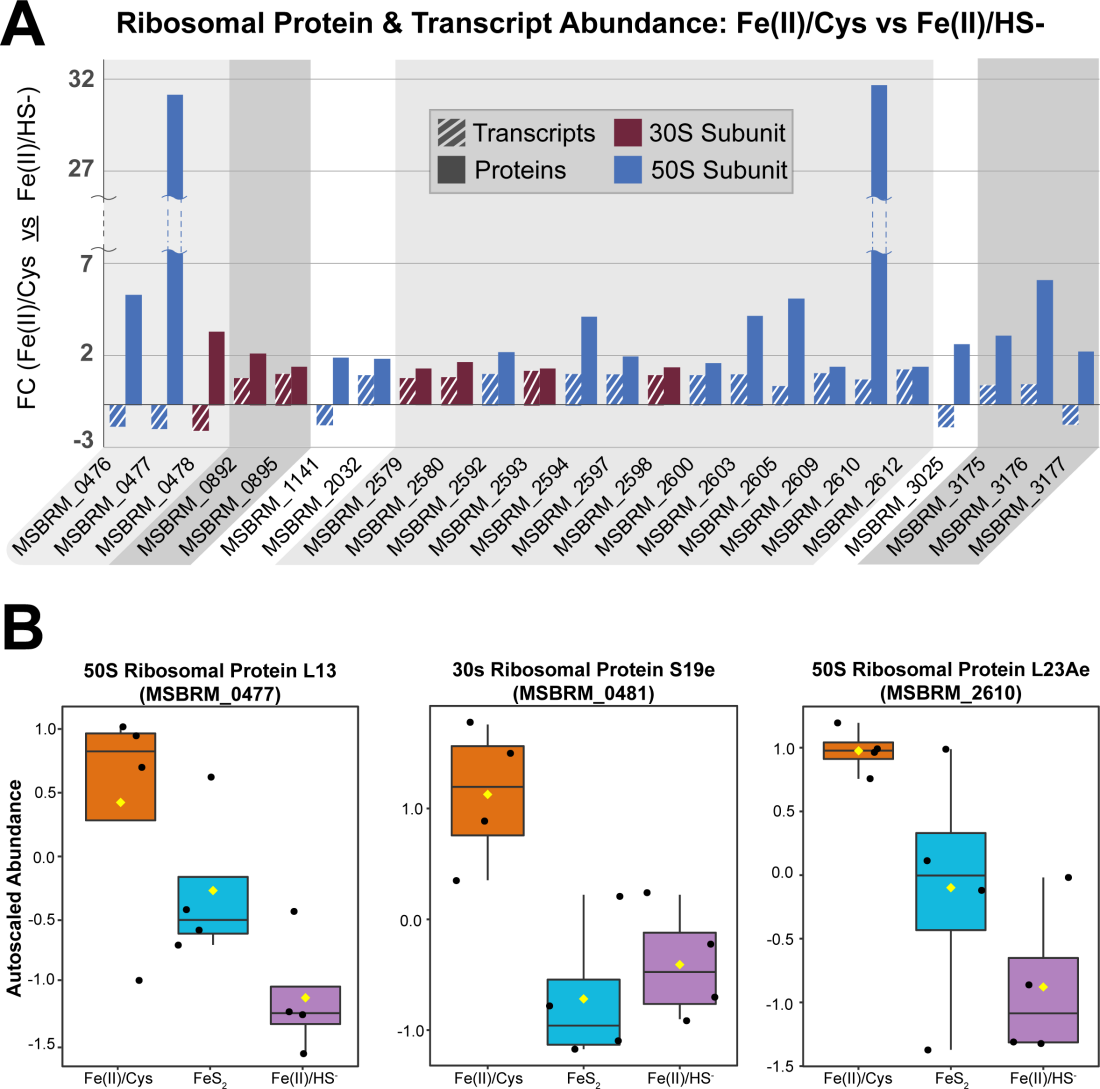
Ribosomal protein and transcript regulatory patterns. (A) Protein (solid bar) and transcript (striped bar) FC of all ribosomal proteins (FDR-adjusted *P* < 0.1) when comparing Cys to HS. Ribosomal protein membership; 50 s subunit (blue) and 30 s subunit (red). Grayscale color blocks distinguish gene operons. (B) Box and whisker plots showing auto-scaled abundance measurements of three ribosomal proteins displaying interesting regulatory patterns (30s RP S19e, 50S RP L13Ae, and 50S ribosomal protein L23Ae).

Ribosomal protein regulation appears to play a large part in the phenotypic differences seen in changing Fe and S sources, with over 30 ribosomal proteins showing measurable alteration. The FeS_2_-grown cells are in between the regulatory patterns of Fe(II)/HS^−^ and Fe(II)/Cys conditions, with ribosomal protein abundance of Fe(II)/Cys > FeS_2_ > Fe(II)/HS^−^. Table S2 shows all statistically significant ribosomal proteins from each two-group comparison, their fold change, and accession information. Interestingly, there was only one ribosomal protein displaying differential abundance with statistical significance in all three comparisons, 50S ribosomal protein L1. The other ribosomal proteins were not shared between the comparisons, indicating that the regulatory pattern is likely due to specific regulation events rather than a general response (Fig. 5). 50S ribosomal protein L1 is involved in translational regulation in addition to protein synthesis, providing additional evidence that translational regulation is crucial to the adjustment for growth on the different substrates. A difference in regulation between the two ribosomal subunits is apparent when comparing FeS_2_ to the other two growth conditions. All 50S ribosomal proteins followed the aforementioned hierarchical abundance pattern Fe(II)/Cys > FeS_2_ > Fe(II)/HS]; however, several 30S ribosomal proteins did not. No 30S subunit ribosomal proteins changed when comparing FeS_2_ to Fe(II)/HS^−^, but five 50S proteins did. The overall difference in ribosome biology was even more evident when comparing Fe(II)/Cys to Fe(II)/HS^−^ where there was large-scale overall changes observed; however, the small and large subunits display similar patterns. Seventeen of 22 large subunit and 8 of 20 small subunit ribosomal proteins were more abundant in cells grown on Fe(II)/Cys, with none being downregulated.

Along with changes in protein concentration, the mRNA transcripts that encode ribosomal proteins also fluctuated between growth conditions. Ribosomal proteins are generally organized in operons and display similar regulatory patterns. Of particular interest are 13 ribosomal proteins that are adjacent in the genome (MSBRM_2579→MSBRM_2612). These 13 ribosomal proteins shared similar mRNA expression profiles, but their proteomic abundance differences were marked. The 50S ribosomal protein L23Ae (MSBRM_2610) stands out in particular, with its protein abundance 32-fold higher in Fe(II)/Cys than Fe(II)/HS^−^, but the transcripts encoding the protein were only upregulated 1.4-fold (Fig. 5). Another large subunit ribosomal protein, L13Ae (MSBRM_477), had a similar regulatory pattern [31.5-fold more abundant in Fe(II)/Cys than Fe(II)/HS^−^]; however, the transcripts decreased in abundance. Two other ribosomal proteins adjacent to L13Ae in the genome shared this pattern of regulation: L18e (MSBRM_0476) and S16e (MSBRM_0478). L23Ae binds to the 23S rRNA and is one of the proteins that rims the polypeptide exit tunnel on the exterior of the ribosome. L13Ae is a structural protein found toward the interior of the ribosome. It is interesting that these structural ribosomal proteins have such a strikingly higher abundance in cells grown with Fe(II)/Cys, especially considering the patterns seen in their respective transcriptional profiles. This could indicate they are involved in more than just ribosome formation and structure and may have moonlighting functions, as is observed in higher organisms (20, 21).

### Methanogenesis and carbon metabolism

In *M. barkeri*, methanogenesis is central to cellular catabolism. Central carbon and methane metabolism showed complete overlap in KEGG when comparing the proteins of interest; therefore, these two categories were combined for the remainder of the analysis. There were large absolute differences in enzymes involved in carbon metabolism between groups, with 24 found to be statistically significant between Fe(II)/Cys and Fe(II)/HS^*−*^ and seven between FeS_2_ and Fe(II)/HS^−^. The phenotype associated with growth on Fe(II)/Cys was particularly unique in its central carbon metabolism. When comparing central carbon enzymes between Fe(II)/Cys and Fe(II)/HS^−^, 15 were less abundant and nine were more abundant in Fe(II)/Cys. Six of the nine more abundant proteins have [Fe-S] clusters, and four are crucial in acetate-driven methanogenesis. Many proteins in higher abundance in the Fe(II)/HS^−^ phenotype are directly or tangentially involved in methanogenesis from CO_2_, and only three bind Fe in any capacity ([Fe-S] cluster or otherwise). Similar patterns were present when comparing Fe(II)/Cys to FeS_2_. Twenty-one proteins were measurably different between these groups. The contrast in [Fe-S] cluster binding is greater in this case, with 6 of 10 proteins in greater abundance in Fe(II)/Cys having [Fe-S] clusters. No central carbon proteins that had a greater abundance in FeS_2_ were annotated as binding [Fe-S] clusters or Fe. Interestingly, 9 out of the 11 proteins were predicted to bind other metal ions, including cobalt, manganese, and magnesium.

Along these lines, FeS_2_ and Fe(II)/HS^−^ experimental groups looked similar with respect to their central carbon and methanogenesis metabolism. Only seven proteins were statistically significant in their differential abundance, four of which were greater in Fe(II)/HS^−^ and three in FeS_2_. Every protein found to be more abundant in Fe(II)/HS^−^ is predicted to bind metal ions. Three of these have auxiliary functions in glycolysis/gluconeogenesis, indicating Fe(II)/HS^−^-grown cells could be subtly modulating their energetics relative to FeS_2_-cultured cells. The three proteins higher in abundance in FeS_2_ are all annotated as methanogenesis enzymes, though they appear to be tangential/secondary to this process. Each is involved in a separate pathway, whose endpoint are all cofactors used by other enzymes to generate methane and energy (methanofuran, coenzyme F_420_ and coenzyme B). Taken together, this provides evidence that the FeS_2_ and Fe(II)/HS^−^ phenotypes are similar in their energetics, especially when compared with Fe(II)/Cys cultures, but still possess subtle differences related to methanogenesis.

**Fig 6:**
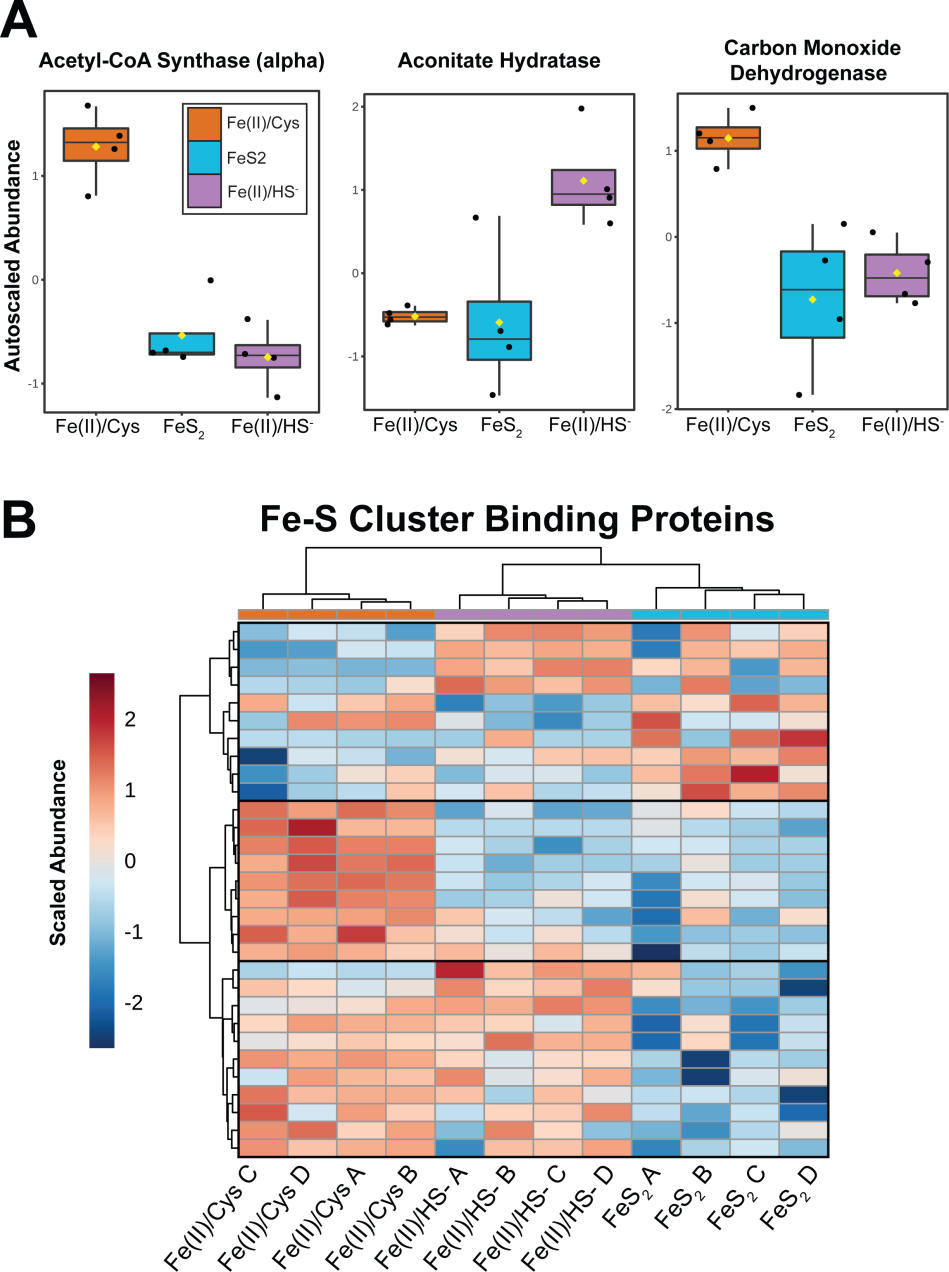
Regulation of central carbon metabolism iron-sulfur binding proteins. (A) Box and whisker plots showing auto-scaled abundance of three relevant carbon metabolism enzymes. Proteins were selected to highlight differential regulation patterns. (B) Hierarchical clustering heatmap of the top 30 (filtered by FDR-corrected *P* -value) iron-sulfur-binding proteins. Clusters 1–3 from the HCA represent three classes of enzymes: (i) cofactor assembly and miscellaneous Fe-S cluster-binding proteins, (ii) carbon metabolism-related enzymes, and (iii) methanogenesis and cofactor biosynthesis proteins.

In general, cells grown on Fe(II)/Cys had a higher abundance of key central carbon enzymes, such as acetyl-CoA synthase and carbon monoxide dehydrogenase, relative to the other two groups (Fig. 6A). This is a general trend, with exceptions such as aconitate hydratase which is upregulated in the Fe(II)/HS^−^ condition (Fig. 6A). This can potentially be attributed to the additional source of organic carbon in the Fe(II)/Cys relative to the others. Cys is converted to alanine during sulfur extraction, which can be used downstream as an input to the central carbon cycle (22, 23). An intriguing pattern emerges when transcriptional regulation of these proteins is compared with protein abundance. The general trend in the proteomics data set, a higher abundance in Fe(II)/Cys-grown cells relative to the other conditions, is mirrored in mRNA regulation patterns for acetyl-CoA synthase and carbon monoxide dehydrogenase. The transcriptomic regulation of aconitate hydratase, however, flipped entirely. Transcripts of this protein were lowest in abundance in the Fe(II)/HS^−^ condition indicating a disconnect between the transcriptome and proteome with respect to aconitate metabolism (16) (Fig. S5).

These data also indicate cells grown on Fe(II)/Cys have a higher abundance of [Fe-S]-binding proteins in their central metabolism, while the other two groups trend toward proteins that bind metal ions (often predicted to be other than Fe). When the proteomics data set was filtered to only include [Fe-S] cluster-containing proteins, a parallel HCA mirrored results from the entire data set with more distinct clustering patterns (Fig. 6B). Clusters formed based on protein function, with a strong high-abundance pattern of central carbon enzymes seen in Fe(II)/Cys, likely due to that condition’s additional source of organic carbon. Other types of Fe-S-binding proteins, like those involved in cofactor biosynthesis or methanogenesis, formed their own, mixed regulation clusters. An uptick in proteins that can perform requisite metabolic functions without [Fe-S] clusters could be metabolically advantageous for cells in [Fe-S] deficient conditions [Fe(II)/HS and FeS_2_]. Metabolic enzymes that utilize metal ions are generally less efficient when compared with those that contain [Fe-S] clusters but in this case could cover some of the metabolic cost associated with the extraction and conversion of insoluble Fe and S from [FeS] aqueous clusters, which are thought to be the form of Fe and S that is assimilated during growth with FeS_2_ (17).

### Metal binding proteins

**Fig 7:**
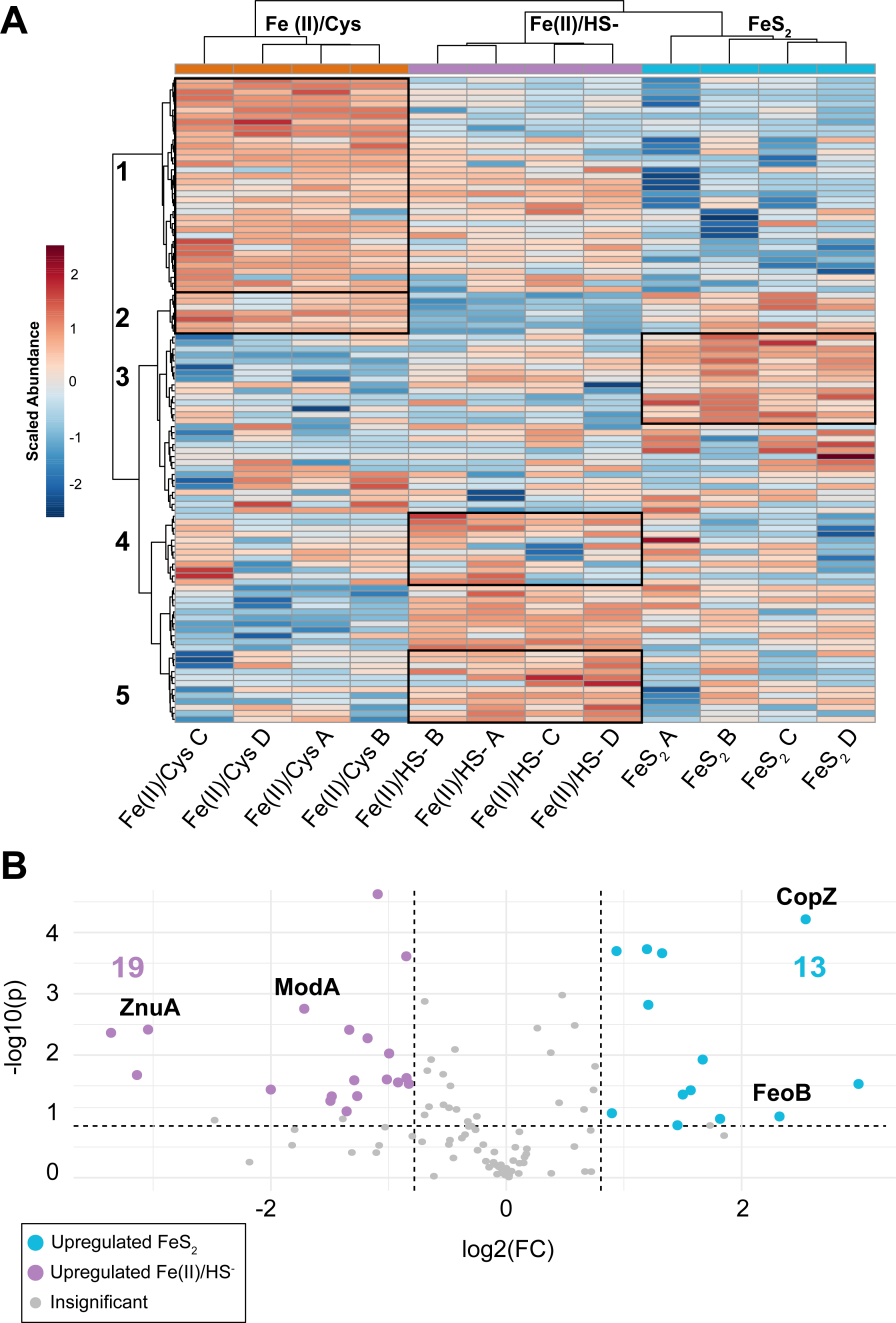
Metal-binding protein differential analysis. (A) Hierarchical clustering analysis of all metal-binding proteins, showing clustering of metal-binding archetypes based on their regulatory patterns. Cluster 1 contains a high proportion of Fe-S cluster binding and carbon metabolism proteins, cluster 2 is comprised of all metal ion-binding motifs, cluster 3 has a low Fe-S cluster-binding representation and heavy metal-binding motifs, cluster 4 is comprised mainly of methane/carbon metabolism proteins, and cluster 5 is mainly Fe-S cluster-binding proteins. (B) Volcano plot of the FeS_2_ vs Fe(II)/HS^−^ comparison. FC is plotted against statistical significance (−log10(*P*)). Nineteen proteins are upregulated in Fe(II)/HS^−^ while 13 are upregulated in FeS_2_. Representative metal-binding proteins are highlighted (copper chaperone CopZ, ferrous iron transporter FeoB, zinc ABC transporter ZnuA, and molybdenum ABC transporter ModA).

To further elucidate patterns in the regulation of metal-binding proteins, a separate statistical analysis was performed that included only proteins predicted to bind metal or alkali metals—Fe, Co, Ni, Cu, Mg, K, Zn, and Mo (107/1019, ~11% of the data set). The DAVID annotation of these proteins revealed that 54 contain [Fe-S] clusters, while the other 53 bind Fe cations or other metal ions. These proteins ranged from the central carbon enzymes discussed above, to copper-binding chaperones (CopZ), translation initiation factors, and metal ion transporters such as the ferrous iron transporter (FeoB). An ANOVA found 59 of the 107 proteins to have statistically significant abundance differences (FDR-adjusted *P* < 0.05), a larger proportion (55%) compared with the unfiltered data set (30%). Of the 59 proteins changing in abundance, 26 bind [Fe-S] clusters, 16 bind magnesium ions, and 6 bind zinc (the remaining bind Co (3), Ni (3), Cu (2), Mn (1), Mo (1), and K (1). A parallel HCA heatmap showed perfect clustering of experimental groups, with large clusters of proteins changing in each group (Fig. 6A). The HCA clustered proteins into regulatory blocks that tended to be composed of proteins with related functions. Cells grown with Fe(II)/Cys had two groups of upregulated proteins, one containing central carbon enzymes and the other metal ion binding proteins. Likewise, Fe(II)/HS^−^-grown cells had two clusters: one containing methanogenesis enzymes while the other contained predominantly [Fe-S] cluster-binding proteins that are not involved in carbon catabolism. The cluster unique to FeS_2_-grown cells contained upregulated proteins that are notably absent of [Fe-S] cluster-binding proteins. This protein cluster was instead enriched with copper binding, cofactor biosynthesis, and methanogenesis enzymes that bind metal ions (Fig. 7A). When the [Fe-S] cluster-binding proteins were analyzed on their own, close to half (29/54) were both statistically relevant (*P* < 0.05) and in higher abundance (FC > 2) in Fe(II)/Cys-grown cells when compared with those grown with Fe(II)/HS^−^ and FeS_2_. This indicates that the [Fe-S] cluster pattern observed in carbon metabolism enzymes is *not* echoed throughout the remainder of the data set—the FeS_2_ and Fe(II)/HS^−^ phenotypes did not show system-wide downregulation of [Fe-S]-binding proteins. This can be clearly seen in the heatmap of all [Fe-S] cluster-binding proteins (Fig. S6). In the Fe(II)/Cys growth condition, [Fe-S]-binding proteins that were upregulated relative to the other groups are concentrated in the carbon metabolic pathway. This provides further evidence of a metabolic shift by the FeS_2_ and Fe(II)/HS^−^ phenotypes; they may be utilizing available [Fe-S] clusters in other biochemical pathways.

There were several metal-binding proteins of interest that showed peculiar regulation patterns. This was the case when comparing FeS_2_ to Fe(II)/HS^−^. A volcano plot of the two-group *t*-test showed a moderate to large statistical difference between the groups in their metal-binding proteins (Fig. 7B). This showed that 31 proteins change, with 13 upregulated in FeS_2_ and 19 in Fe(II)/HS^−^. The iron sulfur assembly protein, SufB, that utilizes cationic Fe and sulfane S liberated from cysteine by SufS/IscS (cysteine desulfurase) (12) was upregulated in both Fe(II)/Cys- and FeS_2_-grown cells relative to those grown with Fe(II)/HS^−^. SufB’s partner ATPase (SufC) showed insignificant abundance changes across all groups. The protein responsible for transporting cationic ferrous iron, FeoB, was also upregulated in FeS_2_, in this case relative to both Fe(II)/Cys and Fe(II)/HS^-^ (Fig. S7). Based on the identified proteins in this analysis, FeS_2_-grown cells appeared to be upregulating the canonical [Fe-S] cluster synthesis pathway relative to Fe(II)/HS^−^, and looks similar to Fe(II)/Cys in terms of certain metal-binding proteins. This could indicate a difference in the abundance of Fe cations in the FeS_2_ growth condition relative to Fe(II)/HS^−^, whether it be through the reductive dissolution process or through active Fe(II) liberation from [FeS] aqueous clusters once assimilated.

Several proteins related to metal transport, scavenging, and chelation are also changing between the groups. Iron (III) dicitrate-binding protein, which binds the ferric iron siderophore dicitrate, shows upregulation in Fe(II)/HS^−^ and FeS_2_. This could be due to cells sensing Fe limitation in these growth conditions, as has been hypothesized previously (13, 17). In *Escherichia coli*, the iron (III) dicitrate-binding protein is part of the five-protein iron dicitrate transport operon, which provides the means for chelated iron (III) to be transported across the membrane. Despite the similarity in name, this protein shows no sequence similarity to FecR, the analogous periplasmic iron (III) dicitrate-binding protein in *E.coli* (24). The protein contains an iron siderophore/cobalamin periplasmic-binding domain (Prosite PS50983) and shares sequence similarity with iron (III) dicitrate-binding proteins across *M. barkeri* strains as well as other *Methanosarcina* species. While it is possible this protein binds iron dicitrate, it could also bind a chelated form of iron related to the [FeS] aqueous clusters or their breakdown products, since it is at similar abundance in Fe(II)/HS^−^ and FeS_2_ growth conditions. CobN-like chelatase BtuS, while less well characterized, appears to have a similar function in metalloporphyrin salvaging. A 2019 study found that *Bacteroides fragilis* required two copies of BtuS to extract essential iron from heme and subsequently transport cationic iron using FeoAB (25). Like *B. fragilis*, we have identified two copies of BtuS (MSBRM_0518 and MSBRM_1640), both of which are upregulated in Fe(II)/Cys and FeS_2_ relative to Fe(II)/HS^*−*^. Due to a lack of the entire operon seen in other organisms such as *B. fragilis*, it is likely the BtuS proteins here are involved in a reappropriation of Fe from heme to other sources as opposed to heme importation, since *M. barkeri* is known to utilize heme as a cofactor in several enzymes including [NiFe]-hydrogenases (12). A related enzyme, siroheme decarboxylase, is involved in the synthesis of heme from iron-porphyrin complexes and also shows an upregulatory pattern in FeS_2_ relative to Fe(II)/HS^−^ (Fig. S7). This could mean the FeS_2_ phenotype is deficient in heme, relative to the Fe(II)/HS^−^ condition, and is compensating by upregulating related biosynthesis pathways.

As observed in other functional categories, transcriptional patterns show mixed correlation with protein abundance. FeoB, for example, has identical transcriptional and protein abundance regulations. Other crucial proteins involved in [FeS] cluster assembly are not regulated similarly between the protein and transcriptional level—the transcripts representing SufB and IscS show marked differences to their respective proteins (Fig. S8). In fact, SufB essentially reverses the order of abundance in the experimental groups, with Fe(II)/HS^−^ cells having the highest number of transcripts followed by Fe(II)/Cys and then FeS_2_. The rampant disconnect between regulatory tiers (e.g., protein vs transcript abundance) has prompted a deeper investigation using metabolomic analyses to help establish directionality of observed regulatory patterns.

### Porphyrin biosynthesis

Porphyrins are key intermediates in the synthesis of many essential cofactors, such as vitamin B_12_ and coenzyme F_430_, in addition to heme and other metal chelators. The Fe(II)/HS^−^ condition stands out when compared with the other groups for its regulation of porphyrin biosynthesis proteins, particularly those involved with cobalamin production. Fe(II)/HS^−^ has seven of eight porphyrin synthesis proteins in lower abundance when compared with both Fe(II)/Cys and FeS_2_. Four of these proteins are shared between the two comparisons (CobB-like chelatase, siroheme dexarboxylase MSBRM_0433, L-threonine 3-o-phosphate decarboxylase, and porphobilnogen deaminase). These four are not adjacent in the related biochemical pathways, but porphobilibogen deaminase and the siroheme decarboxylase are in the same gene neighborhood (MSBRM_0428 and MSBRM_0433, respectively).

L-Threonine 3-o-phosphate decarboxylase and adenosylcobinamide-phosphate synthase are directly adjacent in the genome (MSBRM_0840, MSBRM_0841), and both are much more abundant in Fe(II)/Cys than in Fe(II)/HS^−^ and FeS_2_. These two proteins share D-1-aminopropan-2-ol O-phosphate as a product and substrate, respectively. This molecule is an intermediate between L-threonine and vitamin B_12_, which could be evidence for Fe(II)/Cys-grown cells shuttling L-threonine (and other amino acids) toward vitamin B_12_ or other porphyrin-containing cofactors. Considering cells grown on Fe(II)/Cys only have access to exogenous Fe^2+^, they could be upregulating chelators to better store and shuttle cationic Fe.

FeS_2_ and Fe(II)/Cys appear relatively similar in their porphyrin metabolisms. FeS_2_ has proteins in higher abundance related to the synthesis of heme, while Fe(II)/Cys (along with Fe(II)/HS^−^) has upregulated proteins connected to the interconversion of amino acids to vitamin B_12_.

### Cell-cell recognition/biofilm formation

Another important category that shows differences in regulation is cell-cell recognition/quorum sensing. Cell-cell recognition, generally, is how microorganisms communicate with one another, whether it be intra- or interspecies. Numerous proteins annotated as ABC transporters were found to be changing between growth conditions, namely, the periplasmic binding subunits of dipeptide, oligopeptide, and nickel transporters. Other proteins that share this annotation are related to biofilm formation and virulence, though no *Methanosarcina* species have been observed to display virulent properties. Obvious patterns emerge in the data when comparing Fe(II)/Cys, Fe(II)/HS^−^, and FeS_2_ groups. Fe(II)/Cys has much lower abundances of all ABC transporters involved in cell-cell recognition, with fold changes beyond 130-fold, when compared with FeS_2_. This pattern is echoed when comparing Fe(II)/Cys to Fe(II)/HS^−^: all predicted cell-cell recognition ABC transporters are lower in abundance. There are a total of six separate proteins that are identified as Oppa, the periplasmic binding subunit of the oligopeptide ABC transporter. These six annotations have different sequences and are located away from one another on the genome, with a few exceptions (MSBRM_0219/0220, MSBRM_0900/0901). The pairs of Oppa genes that are adjacent share remarkably similar regulation patterns and have nearly identical sequences, while other proteins distant in the genome are regulated much differently and have dissimilar sequences. The difference in sequence could mean that the proteins have different oligopeptide ligands. Oppa is usually lipid anchored in the membrane, but studies have reported that a soluble form actively binds peptides in the cytosol of other organisms (26). Our collaborators have previously noted that *Methanococcus voltae* (a close cousin to *M. barkeri*) cells grown on FeS_2_ show increased biofilm formation and clumping (14), and the proteomes of Fe(II)/HS^−^- and FeS_2_-grown *M. barkeri* have upregulated proteins related to biofilm formation and signaling. ABC transporters, as a family, are notoriously over-specific in their sequence-based annotation, specifically related to their predicted substrates. The actual substrate of the transport proteins discussed here may vary, and this remains unvalidated, though it is likely the ABC transporters are related to the formation of biofilms irrespective of their actual substrates. Perhaps the [FeS] aqueous clusters generated during the reduction of FeS_2_ act as a stimulus for biofilm-related processes, such as cell clumping and the production of extracellular polymeric substance, the latter of which has been observed in SEM images of *M. barkeri* adhered to FeS_2_ (16).

## DISCUSSION

*Methanosarcina barkeri* MS and Fusaro have both recently been shown to satisfy their Fe and S requirements by reducing FeS_2_ and assimilating reduction products (12–17), breaking a longstanding paradigm that Fe and S are biologically inaccessible in FeS_2_. Our comparative proteomics investigation sought to begin to elucidate the mechanism by which FeS_2_ mineral reduction occurs, how dissolution products are assimilated and trafficked, and how these together influence cellular metabolism in cells provided with different sources of Fe and S. Three growth conditions [Fe(II)/HS^−^, FeS_2_, and Fe(II)/Cys] were employed to probe the effects of soluble and insoluble Fe/S sources as well as the mechanism of exogenous [Fe-S] cluster formation, facilitated either biotically through EET or abiotically (16). We identified over 1,000 proteins, 307 of which showed statistically significant abundance alteration in at least one growth condition. This large ratio of relative abundance change, paired with patterns observed in multivariate statistics, indicates the proteomes from each culturing condition are dramatically different (Fig. 1 and 2). This was evident by a lack of overlap in the PCA, perfect clustering in both HCAs, and the diversity of function in top proteins.

Biochemical pathway analysis is a useful tool to assess exogenous effects on a biological system in a holistic fashion consistent with the scope of proteomics. Proteins were grouped into the pathways they constitute to identify patterns of regulation. Mirroring results from the global multivariate analysis, cells grown on Fe(II)/Cys appear the most different, having generally higher numbers of proteins in each pathway comparison. Cofactor biosynthesis also sticks out as important in the described processes, with more than a dozen proteins altered in each group comparison.

We started the characterization of each phenotype by looking at ribosomal proteins, which were well represented among the differentiated proteins. Much of the ribosomal alteration is consolidated within operons, for example, 13 proteins in the MSBRM_0579 to_0612 gene region which share protein and transcriptional regulation patterns (Fig. 5). The structural ribosomal proteins L23Ae and L13 are among the most drastically changing proteins in the entire data set. Moonlighting activity of analogous ribosomal proteins has been observed in other organisms, notably humans and metazoans (23, 24), raising the possibility they serve similar ribosome-independent functions in *M. barkeri*. Previous studies have noted slight growth rate differences in *M. barkeri* when provided with different sources of Fe and S (17). Consequently, some of the observed changes related to ribosome biology may be attributed to growth kinetics. However, we hypothesize that some of the observed changes to ribosomal proteins (especially those with >15-fold higher abundance) are specific to the changing translational pattern.

As their name would suggest, methanogenesis is often seen as the center point for metabolism in *M. barkeri* and related methanogens. Thus, it is unsurprising to see this category changing in what we are classifying as a Fe- and S-driven phenotypic shift. A pattern in Fe-S cluster binding appears when examining central carbon/methanogenesis pathway changes imparted by Fe/S source differences. Notably, cultures of Fe(II)/Cys have higher abundances of carbon metabolism proteins that bind Fe-S clusters, while the other two groups upregulate metal-ion-binding proteins. This could be due to lower availability of Fe and other thiophilic metals in conditions where HS^−^ is provided or generated via FeS_2_ reduction. Fe(II)/Cys-grown cells also appear to be upregulating central carbon enzymes, which can be explained by their access to an additional source of organic carbon. Since it is the only source of sulfur, Cys is desulfurated by SufS which likely generates an excess of alanine that can be directly converted to pyruvate by an aminotransferase. Therefore, an upregulation of central carbon enzymes would be required to process this influx. There also appears to be changes in methanogenesis source between Fe(II)/HS^−^**-** and Fe(II)/Cys-grown cells that could be a direct result of the metal-binding patterns. Interestingly, the FeS_2_ and Fe(II)/HS^−^ groups look similar in their energetics (based on expression of core methanogenesis pathway proteins). This is in contrast to what we expected given the FeS_2_ group must facilitate additional electron transfers via EET in the reduction of FeS_2_.

Considering the experimental design where some conditions were provided HS^−^ and where HS^−^ could be generated due to FeS_2_ reduction, it is of little surprise that major proteomics differences between the phenotypes were found in proteins that bind metals, in particular those that are thiophilic. The proportion and importance of metal binding proteins in the data set warranted a separate statistical analysis. Key enzymes in the [FeS] cluster synthesis pathways are regulated in this regard including the Fe(II) transporter FeoB, cysteine desulfurase SufS, and iron sulfur cluster assembly protein SufB. This represents the major components of the route for assimilation of Fe(II) and synthesis of [FeS] clusters, and all are higher abundance in FeS_2_ than in Fe(II)/HS^−^. Interestingly, a previous study found that FeS_2_-grown *M. voltae* cells hyperaccumulated Fe and stored it as a thioferrate-like intracellular mineral phase (17). This study too found that FeoB was up-expressed, which was hypothesized to be due to the cells sensing Fe(II) limitation due to it being complexed with HS^−^. This observation also forms the basis for the suggestion that cells are possibly directly assimilating neutrally charged [FeS] aqueous clusters as their source of Fe and S in FeS_2_ growth conditions. The results obtained herein with *M. barkeri*, where FeoB, SufB, and SufS are all up-expressed in FeS_2_-grown cells, are potentially consistent with these cells also incorrectly sensing the limitation of Fe and S [as intracellular (FeS) clusters]. The Fe(II)/Cys and FeS_2_ groups look very similar in their regulation of SufS, the enzyme that frees S from cysteine for use in Fe-S clusters. A metalloporphyrin salvage enzyme (BtuS), which may function in removing Fe from heme typically observed in other organisms experiencing Fe limitation, shows upregulation in Fe(II)/Cys- and FeS_2_-grown cells. Neither group shows stress indicators or attenuated growth indicative of Fe limitation, so it is likely related to heme processing and interconversion to other forms of Fe.

Other categories that help explain the statistical differences between groups, such as quorum sensing and porphyrin biosynthesis, echo the results from the remainder of the investigation. Peptide transporters that may function in biofilm formation and other cell-cell communications varied by over 130-fold abundance in some comparisons. Cell surface proteins with PKD domains that are predicted to mediate extracellular matrix interactions are also regulated in response to the Fe and S environment. The extracellular matrix clearly plays a role in cellular-mineral interactions (16), so the further characterization of these cell surface proteins (CSP) and transporters will aid in elucidating the communicative portion of *M. barkeri*’s ability to form biofilms and colonize the surface of minerals.

This study provides the groundwork for the full elucidation of the mechanism utilized by *Methanosarcina barkeri* to access nutrients (Fe, S) from inert, Earth-abundant iron-sulfur minerals including FeS_2_. This work builds upon many previous analyses, including an analogous investigation on the proteome of *Methanococcus voltae* (27). Steward et al. showed a greater degree of overall abundance changes (40% of identified proteins) in the comparison of *M. voltae* grown on FeS_2_ compared with Fe(II)/HS^−^. Similar to the observations in this study, *M. voltae* showed distinct protein abundance patterns in proteins ranging in function from ribosomal structure components to metal binding and cellular energetics. Several key proteins show similarity in their regulatory patterns, including the increased abundance of FeoB in FeS_2_-grown cells. Comparable multivariate statistics on the proteomes of two organisms from different taxonomic orders is consistent with the importance of Fe and S sources and availability on the phenotype of methanogens (28).

Presented here is a bounty of enzymes and proteins primed for further investigation via knockout studies, direct assays, and more. The top targets include the following: ribosomal proteins changing by more than 30-fold, which are likely to be interacting with the genome or RNAs in a regulatory fashion, peptide transporters related to cell-cell or cell-environment communication, and metal binding and transport proteins like FeoB, SufS, and the iron (III) dicitrate-binding protein. The proteomic evidence presented here suggests that the three Fe and S sources provided induce unique and distinct phenotypes of *M. barkeri*. Transcriptomics data present an interesting, mixed corroboratory relationship with the proteomics analysis. The abundance of some proteins precisely mirrors the pattern seen in the transcripts while others seem either completely flipped or entirely unrelated. Forthcoming metabolomic and lipidomic studies on this system, in these exact growth conditions, will present a holistic view of methanogen biology and will provide grounds to present a model of Fe and S sources and acquisition dynamics in *M. barkeri* and its effects of cell biology.

*Methanosarcina* species, including *M. barkeri*, have evolved to survive in a wide range of habitats, ranging from the mammalian gut to freshwater and marine sediments (29). It is a clear advantage to have a broad toolset for assessing that Fe and S from a variety of sources, whose abundance likely varies based on habitat. This study suggests *M. barkeri* achieves this from the ground up, changing its entire cellular makeup to compensate for changes in just two elements, Fe and S. Considering that FeS_2_ is host to a number of other thiophilic elements [i.e., Co and Ni (30)] that are also necessary and highly utilized by a variety of methanogens (31), developing a better understanding of how this organism extracts elements from FeS_2_ will provide a foundation for developing novel bio-mining technologies and will also provide new insights into how such metals are acquired in anoxic and euxinic environments where FeS2 is likely the predominant source of such elements.

## MATERIALS AND METHODS

### Cell cultivation

*M. barkeri* strain MS was obtained from the American Type Culture Collection (ATCC-43569). Cells were grown following previously described methods (16) in anoxic, low-salinity medium and without added Fe or S. Cultures for proteomics analysis were grown with three different Fe and S sources: (i) 2 mM cysteine and 20 µM FeCl_2_, (ii) 2 mM Na_2_S and 20 µM FeCl_2_, or (iii) 2 mM synthetic nanoparticulate FeS_2_ prepared as previously described. When cultures reached mid-log phase, as determined by CH_4_ and DNA production monitored using previously described methods (13), cells were harvested under an anoxic headspace by centrifugation at 4,600 × *g* for 30 minutes at 4°C. The supernatant was then decanted in an anaerobic chamber before submerging the cell pellet in liquid N_2_ to flash freeze. Frozen cell pellets were stored at −80°C until further processing for proteomics.

### Proteomics sample preparation

Cultures were prepared for ongoing multi-omics analysis where proteins, metabolites, and lipids were extracted in parallel from the same cell pellets for this study, and a future study focused on metabolomics and lipidomics. For cell lysis, cell pellets were resuspended in 5 mL 50 mM ammonium bicarbonate (NH_4_HCO_3_) with a protease inhibitor mix at pH 8 (Complete Mini EDTA Free Protease Inhibitor Cocktail, Roche). Samples were lysed using an ultrasonic homogenizer at 40% power output for 15 minutes. Resulting lysate was centrifuged at 4,000 × *g* for 15 minutes at 4°C. The supernatant was isolated for soluble protein preparation. Protein concentration was estimated using nanodrop spectrophotometer (Thermo-Fisher), and a volume equivalent to 100 µg of protein was aliquoted. For reduction and alkylation, DTT was added to a final concentration of 5 mM and incubated at room temperature for 30 minutes. Iodoacetamide was added to a final concentration of 15 mM, and the mixture was placed in the dark for 30 minutes at room temperature. For filter-assisted tryptic digest, a filter-assisted trypsin digestion was performed on a 3-kDa spin filter at 1:20 m/m trypsin:protein overnight at 37°C. Trypsin was quenched through the addition of TFA (0.1%) and spun to filter tryptic peptides.

### Shotgun proteomics data acquisition

Tryptic peptides were cleaned and concentrated using a C18 desalting column and were separated by reverse-phase XSelect CSH C18 2.5 µm resin (Waters) on an in-line 150 × 0.075 mm column using an UltiMate 3000 RSLCnano system (Thermo). Peptides were eluted using a 90-minute gradient from 98:2 to 65:35 buffer A:B ratio (Buffer A = 0.1% formic acid, 0.5% acetonitrile; Buffer B = 0.1% formic acid, 99.9% acetonitrile). Eluted peptides were ionized by electrospray (2.4 kV) followed by mass spectrometric analysis on an Orbitrap Eclipse Tribrid mass spectrometer (Thermo-Fisher). MS data were acquired using the FTMS analyzer in profile mode at a resolution of 120,000 over a range of 375 to 1,200 m/z. Following HCD activation, fragment MS spectral (fMS/MS) data were acquired using the ion trap analyzer in centroid mode and normal mass range with a normalized collision energy of 30%. Proteins were identified by database search using MaxQuant (Max Planck Institute) label-free quantification with a parent ion tolerance of 2.5 ppm and a fragment ion tolerance of 0.5 Da. Scaffold Q+S (Proteome Software) was used to verify fMS/MS-based peptide and protein identifications using strict trypsin specificity including shared peptides. Protein identifications were accepted if they could be established with less than 1.0% false discovery and contained at least 2 identified peptides with a minimum length of 7 AA. Modifications specified were carbamidomethyl (C) (fixed), oxidation (M) (variable), acetyl (Protein N-term) (variable). Protein probabilities were assigned by the Protein Prophet algorithm. All protein identifications were based on a reference genome acquired from NCBI-BioProject (accession no. PRJNA230939) which encodes for open reading frames (protein sequences).

### Statistical, functional, and pathway analyses

Data from Scaffold Q+S represent extracted protein intensities from the top three peptides (based on confidence in the identification) for each protein. Raw data were exported from scaffold and uploaded to MetaboAnalyst 5.0 (20, 21) for statistical analysis. Data underwent a quantile normalization followed by a log_10_ transformation. Data are shown as auto-scaled values (mean centered, divided by standard deviation of each variable), unless otherwise specified. For the global statistical analysis, the ANOVA significance threshold was set to *P* < 0.05. The features in the ANOVA underwent a Fishers least significant difference post-hoc analysis, and all reported *P*-values are FDR corrected. To improve sensitivity for pathway analysis, proteins with *P*-values of up to 0.1 post FDR correction were included in pairwise analyses. Pathway analysis data were then manually curated to remove pathways not supported by proteins with *P* < 0.5. Heatmaps employed Ward’s method of HCA and utilized Euclidian distances. DAVID (18) and KEGG (19) were used for protein characterization, categorization, and pathway analysis.

## Data Availability

Data are available in the MassIVE repository under accession no. PXD042477 (version 1.3.16).

## SUPPLEMENTAL MATERIAL

Figure S1 spectrum.00418-23-s0001.pdf All group multivariate statistics comparison.

Figure S2 spectrum.00418-23-s0002.pdf Box and whisker plots corresponding to the top 35 proteins, filtered by P-value.

Figure S3 spectrum.00418-23-s0003.pdf PLSDA VIP plot of the top 15 proteins, filtered by VIP score.

Figure S4 spectrum.00418-23-s0004.pdf Whole dataset functional distribution characterization.

Figure S5 spectrum.00418-23-s0005.pdf Central carbon metabolism transcriptional regulation.

Figure S6 spectrum.00418-23-s0006.pdf Hierarchical clustering heatmap of all iron-sulfur binding proteins.

Figure S7 spectrum.00418-23-s0007.pdf Regulation of key metal binding proteins.

Figure S8 spectrum.00418-23-s0008.pdf Regulatory patterns of the mRNA transcripts and proteins of 3 key metal binding proteins.

Tables S1 to S3 spectrum.00418-23-s0009.docx Protein identifiers and abundance change of ribosomal proteins.

Tables S4 to S15 spectrum.00418-23-s0010.xlsb All proteins, their annotations, pathway analysis, statistics (ANOVA and two-group), and other figure information.

ASM does not own the copyrights to Supplemental Material that may be linked to, or accessed through, an article. The authors have granted ASM a non-exclusive, world-wide license to publish the Supplemental Material files. Please contact the corresponding author directly for reuse.

## References

[1] Archibald F. 1983. Lactobacillus plantarum, an organism not requiring iron . FEMS Microbiol Lett 19:29–32. doi:10.1111/j.1574-6968.1983.tb00504.x

[2] Beinert H, Holm RH, Münck E. 1997. Iron-sulfur clusters: nature's modular, multipurpose structures. Science 277:653–659. doi:10.1126/science.277.5326.653 9235882

[3] Johnson DC, Dean DR, Smith AD, Johnson MK. 2005. Structure, function, and formation of biological iron-sulfur clusters. Annu Rev Biochem 74:247–281. doi:10.1146/annurev.biochem.74.082803.133518 15952888

[4] Imlay JA. 2006. Iron-sulphur clusters and the problem with oxygen. Mol Microbiol 59:1073–1082. doi:10.1111/j.1365-2958.2006.05028.x 16430685

[5] Ayala-Castro C, Saini A, Outten FW. 2008. Fe-S cluster assembly pathways in bacteria. Microbiol Mol Biol Rev 72:110–125, doi:10.1128/MMBR.00034-07 18322036 PMC2268281

[6] Brzóska K, Meczyńska S, Kruszewski M. 2006. Iron-sulfur cluster proteins: Electron transfer and beyond. Acta Biochim Pol 53:685–691. doi:10.18388/abp.2006_3296 17143336

[7] Pattus F, Abdallah MA. 2000. Siderophores and iron-transport in microorganisms. J Chinese Chemical Soc 47:1–20. doi:10.1002/jccs.200000001

[8] Lau CKY, Krewulak KD, Vogel HJ. 2016. Bacterial ferrous iron transport: the Feo system. FEMS Microbiol Rev 40:273–298. doi:10.1093/femsre/fuv049 26684538

[9] Wu B, Liu F, Fang W, Yang T, Chen G-H, He Z, Wang S. 2021. Microbial sulfur metabolism and environmental implications. Sci Total Environ 778:146085. doi:10.1016/j.scitotenv.2021.146085 33714092

[10] Liu Y, Sieprawska-Lupa M, Whitman WB, White RH. 2010. Cysteine is not the sulfur source for iron-sulfur cluster and methionine biosynthesis in the methanogenic archaeon Methanococcus maripaludis. J Biol Chem 285:31923–31929. doi:10.1074/jbc.M110.152447 20709756 PMC2952193

[11] Major TA, Burd H, Whitman WB. 2004. Abundance of 4Fe–4S motifs in the genomes of methanogens and other prokaryotes. FEMS Microbiol Lett 239:117–123. doi:10.1016/j.femsle.2004.08.027 15451109

[12] Christina J, Alexis E, Mason M-E, CD R, DJ L, BE S, MW W. 2022. Pathways of iron and sulfur acquisition, cofactor assembly, destination, and storage in diverse archaeal methanogens and alkanotrophs. J Bacteriol 203:e00117–21. doi:10.1128/JB.00117-21 PMC8351635 34124941

[13] Spietz RL, Payne D, Szilagyi R, Boyd ES. 2022. Reductive biomining of pyrite by methanogens. Trends Microbiol 30:1072–1083. doi:10.1016/j.tim.2022.05.005 35624031

[14] Payne D, Spietz RL, Boyd ES. 2021. Reductive dissolution of pyrite by methanogenic archaea. ISME J 15:3498–3507. doi:10.1038/s41396-021-01028-3 34112969 PMC8630215

[15] Boyd E, Payne D, Shepard E, Spietz R, Guo Y, Broderick W, Broderick J. 2022. Biomining metals from pyritic ores: physiological, biochemical, and biophysical considerations. Biophys J 121:296a. doi:10.1016/j.bpj.2021.11.1269

[16] Spietz RL, Payne D, Kulkarni G, Metcalf WW, Roden EE, Boyd ES. 2022. Investigating abiotic and biotic mechanisms of pyrite reduction. Front Microbiol 13:878387. doi:10.3389/fmicb.2022.878387 35615515 PMC9124975

[17] Devon P, M. SE, L. SR, Katherine S S, Sue B, Mark Y, Brian B, E. BW, B. BJ, S. BE, Y. GM. 2022. Examining pathways of iron and sulfur acquisition,trafficking, deployment, and storage in mineral-grown methanogen cells. J Bacteriol 203:e00146-21. doi:10.1128/jb.00146-21 PMC8516115 34251867

[18] Sherman BT, Hao M, Qiu J, Jiao X, Baseler MW, Lane HC, Imamichi T, Chang W. 2022. DAVID: a web server for functional enrichment analysis and functional annotation of gene lists. Nucleic Acids Res 50:W216–W221. doi:10.1093/nar/gkac194 35325185 PMC9252805

[19] Kanehisa M, Goto S. 2000. KEGG: Kyoto Encyclopedia of Genes and Genomes. Nucleic Acids Res 28:27–30. doi:10.1093/nar/28.1.27 10592173 PMC102409

[20] Chaudhuri S, Vyas K, Kapasi P, Komar AA, Dinman JD, Barik S, Mazumder B. 2007. Human ribosomal protein L13A is dispensable for canonical ribosome function but indispensable for efficient rRNA methylation. RNA 13:2224–2237. doi:10.1261/rna.694007 17921318 PMC2080596

[21] Qi Y, Li X, Chang C, Xu F, He Q, Zhao Y, Wu L. 2017. Ribosomal protein L23 negatively regulates cellular apoptosis via the RPL23/Miz-1/c-Myc circuit in higher-risk myelodysplastic syndrome. Sci Rep 7:2323. doi:10.1038/s41598-017-02403-x 28539603 PMC5443795

[22] Hidese R, Inoue T, Imanaka T, Fujiwara S. 2014. Cysteine desulphurase plays an important role in environmental adaptation of the hyperthermophilic archaeon Thermococcus kodakarensis. Mol Microbiol 93:331–345. doi:10.1111/mmi.12662 24893566

[23] Mihara H, Esaki N. 2002. Bacterial cysteine desulfurases: their function and mechanisms. Appl Microbiol Biotechnol 60:12–23. doi:10.1007/s00253-002-1107-4 12382038

[24] Welz D, Braun V. 1998. Ferric citrate transport of Escherichia coli: functional regions of the FecR transmembrane regulatory protein. J Bacteriol 180:2387–2394. doi:10.1128/JB.180.9.2387-2394.1998 9573190 PMC107180

[25] Rocha ER, Bergonia HA, Gerdes S, Jeffrey Smith C. 2019. Bacteroides fragilis requires the ferrous-iron transporter FeoAB and the CobN-like proteins BtuS1 and BtuS2 for assimilation of iron released from heme. Microbiologyopen 8:e00669. doi:10.1002/mbo3.669 29931811 PMC6460266

[26] Balestrieri M, Gogliettino M, Fiume I, Pocsfalvi G, Catara G, Rossi M, Palmieri G. 2011. Structural and functional insights into aeropyrum pernix OppA, a member of a novel archaeal OppA subfamily. J Bacteriol 193:620–630. doi:10.1128/JB.00899-10 21097609 PMC3021232

[27] Steward KF, Payne D, Kincannon W, Johnson C, Lensing M, Fausset H, Németh B, Shepard EM, Broderick WE, Broderick JB, Dubois J, Bothner B. 2022. Proteomic analysis of Methanococcus voltae grown in the presence of mineral and nonmineral sources of iron and sulfur. Microbiol Spectr 10:e0189322. doi:10.1128/spectrum.01893-22 35876569 PMC9431491

[28] Liu Y, Beer LL, Whitman WB. 2012. Methanogens: a window into ancient sulfur metabolism. Trends Microbiol 20:251–258. doi:10.1016/j.tim.2012.02.002 22406173

[29] Boone DR, Whitman WB, Koga Y. 2015. Methanosarcinales ord. In Bergey’s Manual of Systematics of Archaea and Bacteria:1–1. doi:10.1002/9781118960608

[30] Hawley JE, Nichol I. 1961. Trace elements in pyrite, pyrrhotite and chalcopyrite of different ores. Economic Geology 56:467–487. doi:10.2113/gsecongeo.56.3.467

[31] Scherer P, Lippert H, Wolff G. 1983. Composition of the major elements and trace elements of 10 methanogenic bacteria determined by Inductively coupled plasma emission spectrometry. Biol Trace Elem Res 5:149–163. doi:10.1007/BF02916619 24263482

