# Supplementary material for: A shift between mineral and nonmineral sources of iron and sulfur causes proteome-wide changes in Methanosarcina barkeri
Source: Microbiol Spectr. 2024 Jan 5;12(2):e00418-23. doi: 10.1128/spectrum.00418-23 (PMC10846266; doi:10.1128/spectrum.00418-23)
Supplement: Figure S1 — All group multivariate statistics comparison. [file spectrum.00418-23-s0001.pdf]

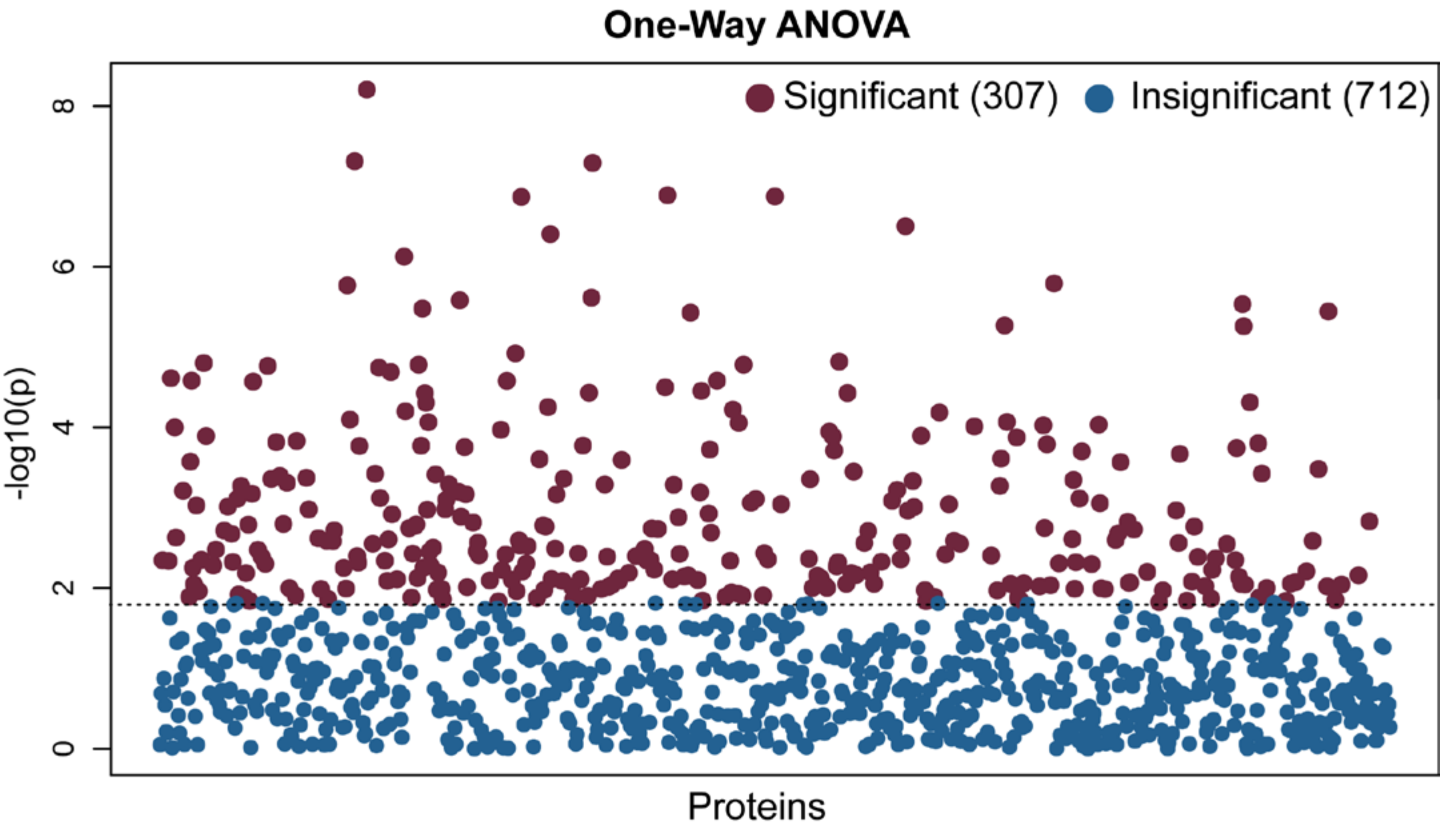

Figure S1. All group multivariate statistics comparison. One-way ANOVA of protein abundance for 1019 identified proteins. Of the total, 307 proteins meet the criteria for statistical significance (FDR Corrected p-value <0.05) (red). The remaining 712 proteins do not meet this criteria (blue).
