# Supplementary material for: A shift between mineral and nonmineral sources of iron and sulfur causes proteome-wide changes in Methanosarcina barkeri
Source: Microbiol Spectr. 2024 Jan 5;12(2):e00418-23. doi: 10.1128/spectrum.00418-23 (PMC10846266; doi:10.1128/spectrum.00418-23)
Supplement: Figure S2 — Box and whisker plots corresponding to the top 35 proteins, filtered by P-value. [file spectrum.00418-23-s0002.pdf]

Figure S2

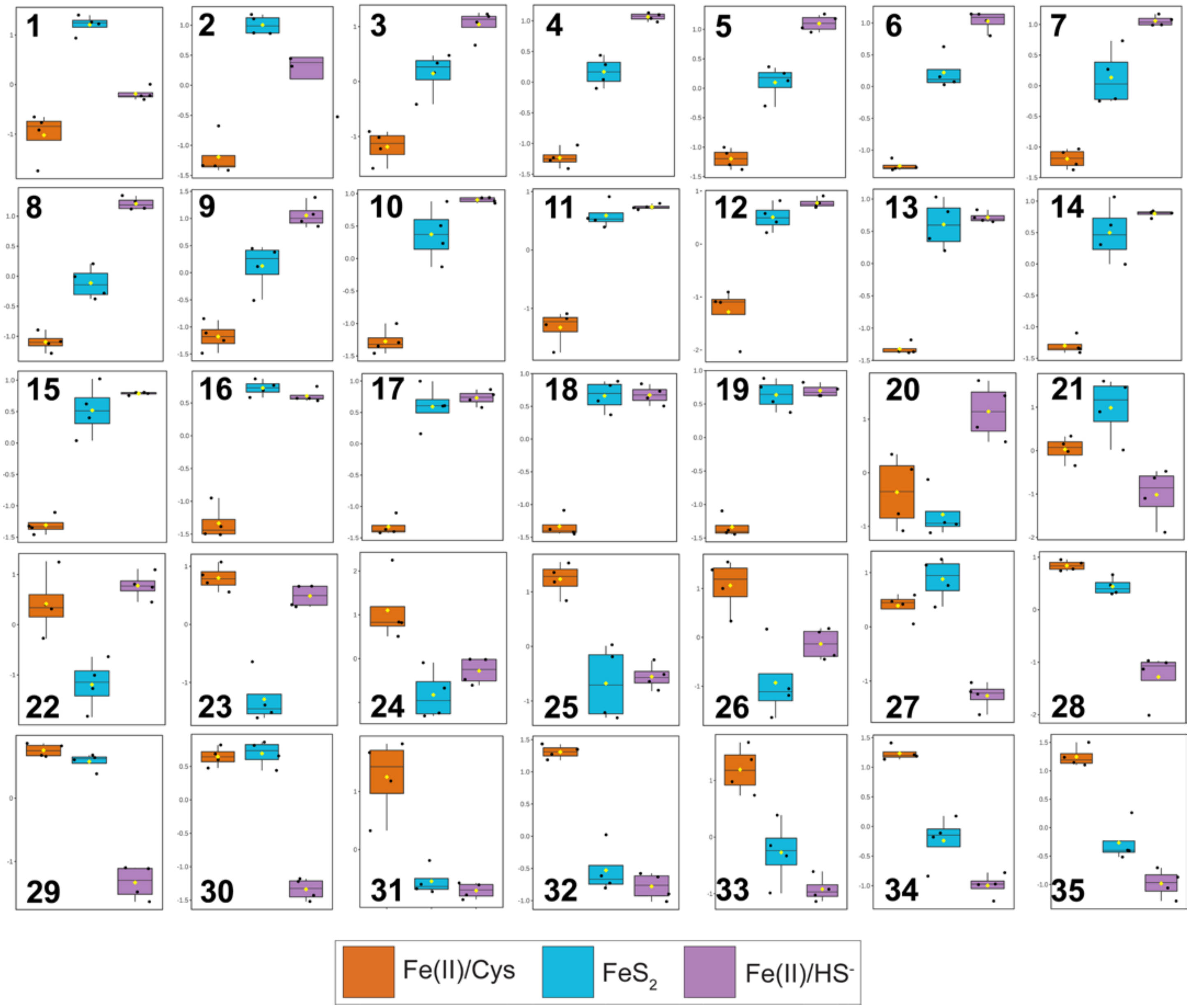

Figure S2. Box and whisker plots corresponding to the top 35 proteins (filtered by FDR corrected p-value). Abundance values are auto-scaled.
