# Supplementary material for: A shift between mineral and nonmineral sources of iron and sulfur causes proteome-wide changes in Methanosarcina barkeri
Source: Microbiol Spectr. 2024 Jan 5;12(2):e00418-23. doi: 10.1128/spectrum.00418-23 (PMC10846266; doi:10.1128/spectrum.00418-23)
Supplement: Figure S3 — PLSDA VIP plot of the top 15 proteins, filtered by VIP score. [file spectrum.00418-23-s0003.pdf]

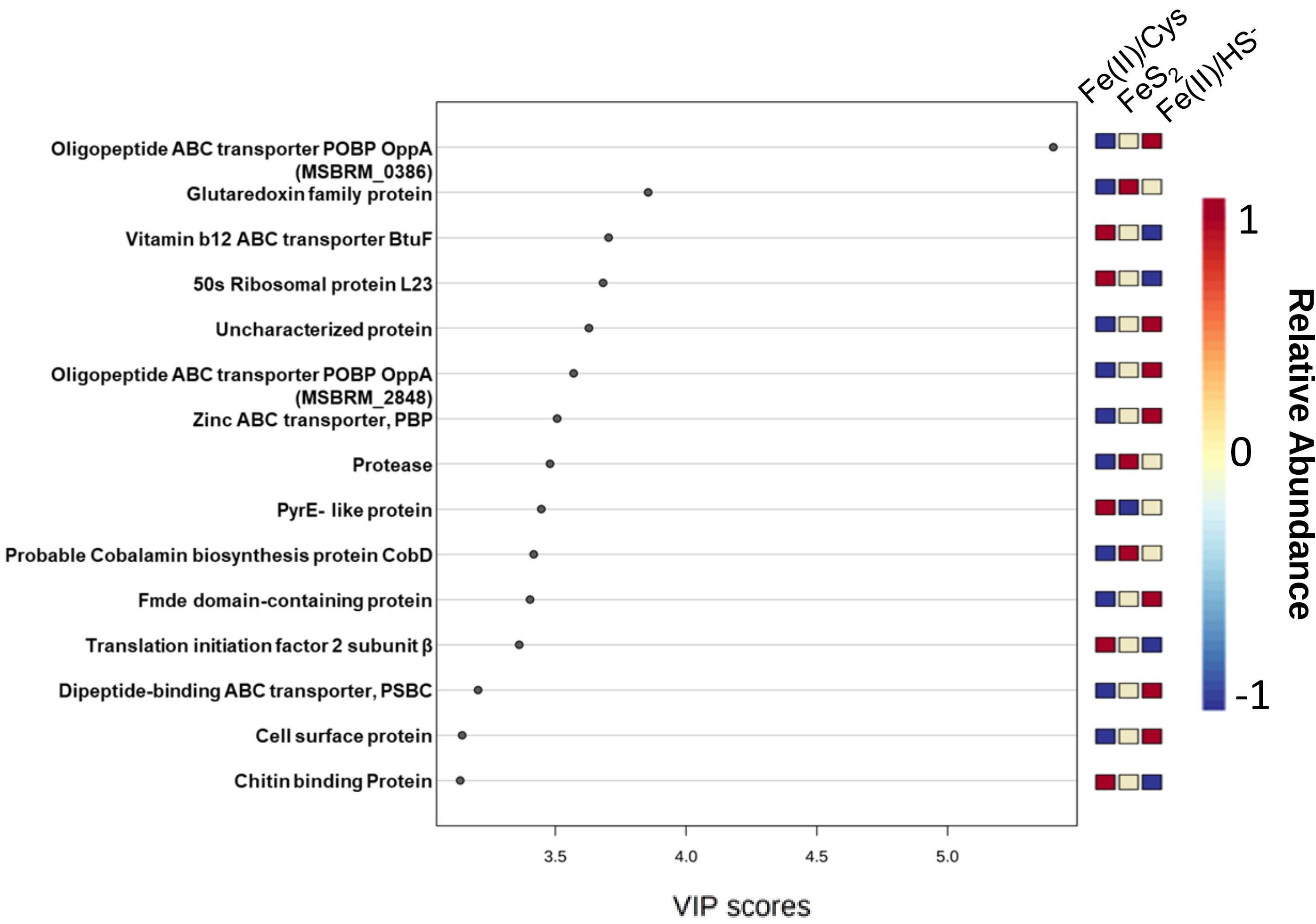

Figure S3: PLSDA VIP plot of the top 15 proteins, filtered by VIP score. Legend on the right displays the relative abundance of the protein in each group. P-values of the listed proteins range from 0.000107 (Chitin Binding Protein) to 2.46E-06 (Oligopeptide ABC transporter POPB OppA MSBRM_0386).
