# Supplementary material for: A shift between mineral and nonmineral sources of iron and sulfur causes proteome-wide changes in Methanosarcina barkeri
Source: Microbiol Spectr. 2024 Jan 5;12(2):e00418-23. doi: 10.1128/spectrum.00418-23 (PMC10846266; doi:10.1128/spectrum.00418-23)
Supplement: Figure S4 — Whole dataset functional distribution characterization. [file spectrum.00418-23-s0004.pdf]

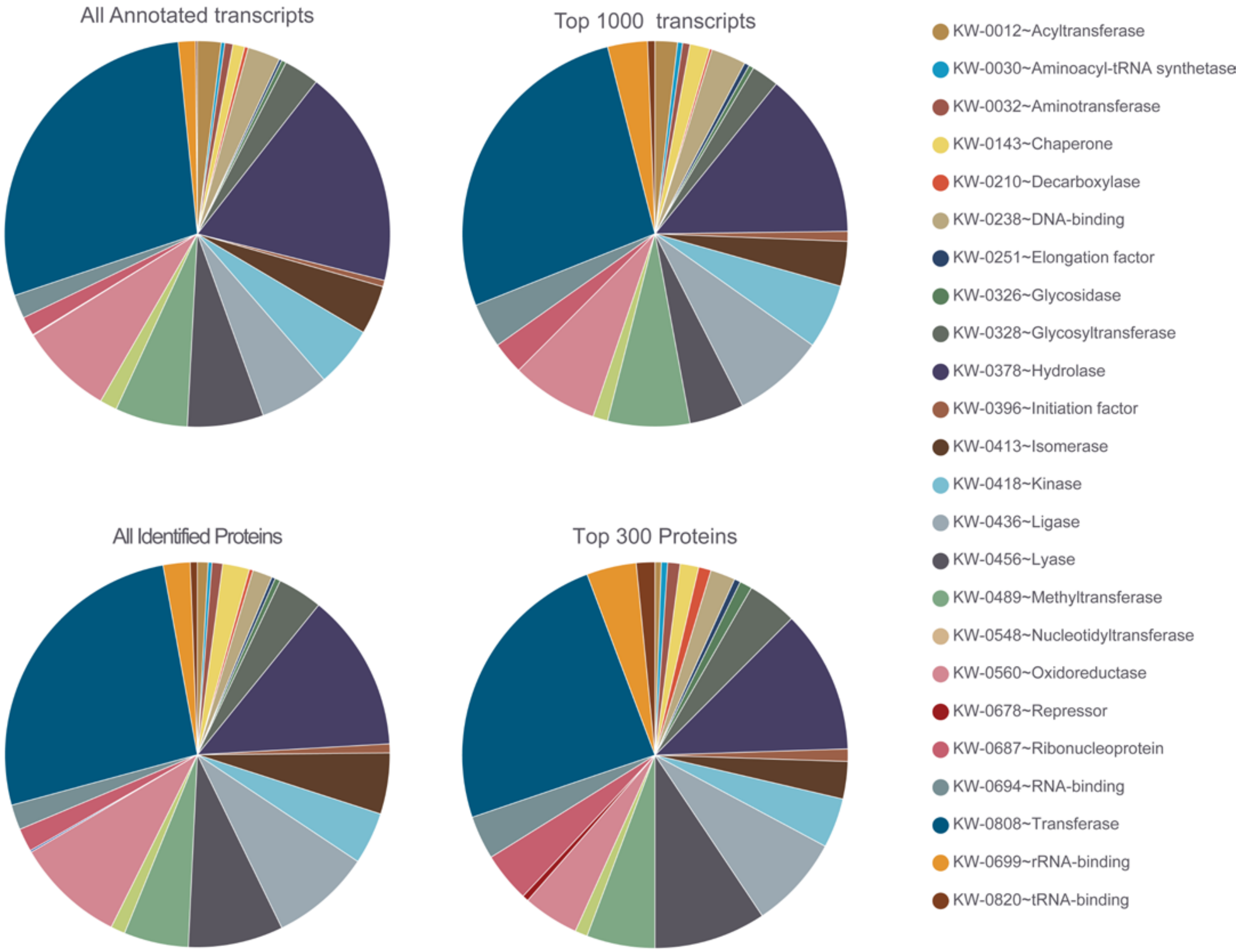

DNA Replication/Transcription	Translation/Chaperone	Energy Metabolism	Secondary Metabolism	Cell Transport/Structural/Misc.	Uncharacterized
14	45	47	93	32	36

Figure S4. Whole dataset functional distribution characterization. A: Functions of encoded proteins of all detected transcripts (3000), B: Top (filtered by FDR corrected p-value) 1000 transcripts, C: All identified proteins and D: Top 300 proteins-filtered by FDR corrected p-value. Transcript number in B was selected to reflect the top 1/3 of the dataset so as to allow comparison to significant proteins (307/1019).
