# Supplementary material for: A shift between mineral and nonmineral sources of iron and sulfur causes proteome-wide changes in Methanosarcina barkeri
Source: Microbiol Spectr. 2024 Jan 5;12(2):e00418-23. doi: 10.1128/spectrum.00418-23 (PMC10846266; doi:10.1128/spectrum.00418-23)
Supplement: Figure S5 — Central carbon metabolism transcriptional regulation. [file spectrum.00418-23-s0005.pdf]

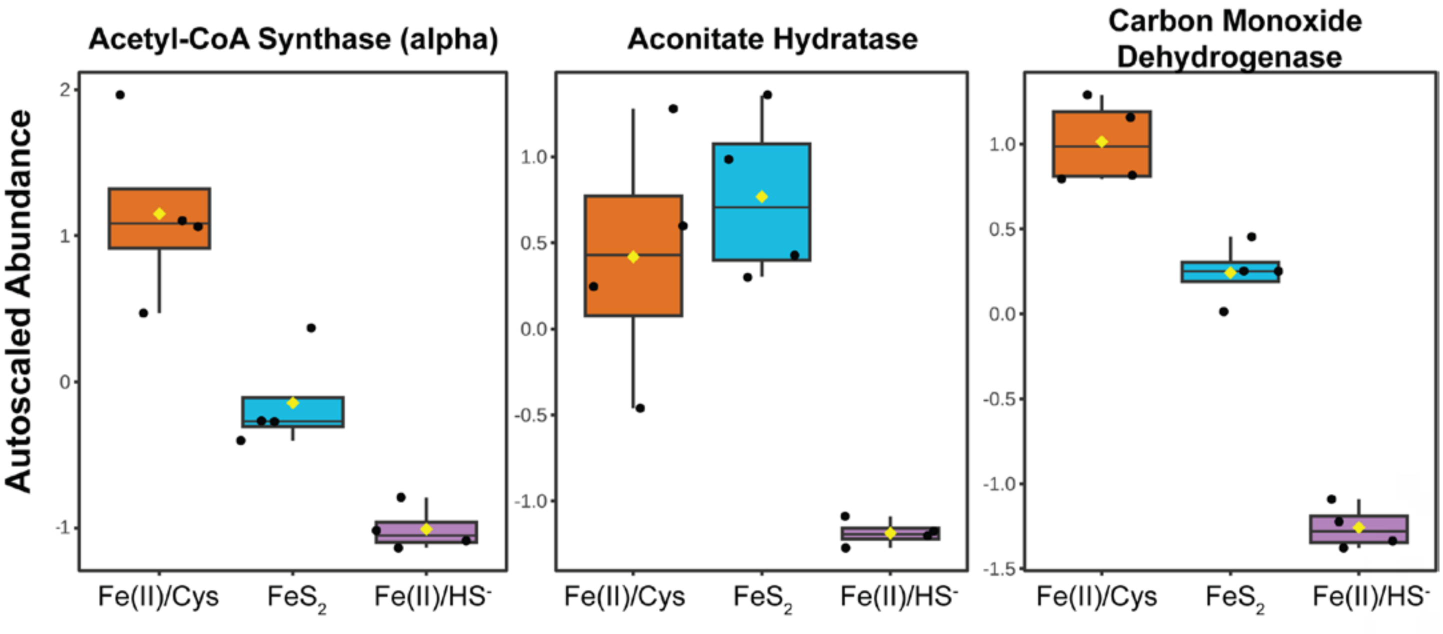

Figure S5. Central carbon metabolism transcriptional regulation. Regulatory patterns of the transcripts of 3 carbon metabolism enzymes. Box and whisker plots show scaled abundance in each condition.
