# Supplementary material for: A shift between mineral and nonmineral sources of iron and sulfur causes proteome-wide changes in Methanosarcina barkeri
Source: Microbiol Spectr. 2024 Jan 5;12(2):e00418-23. doi: 10.1128/spectrum.00418-23 (PMC10846266; doi:10.1128/spectrum.00418-23)
Supplement: Figure S6 — Hierarchical clustering heatmap of all iron-sulfur binding proteins. [file spectrum.00418-23-s0006.pdf]

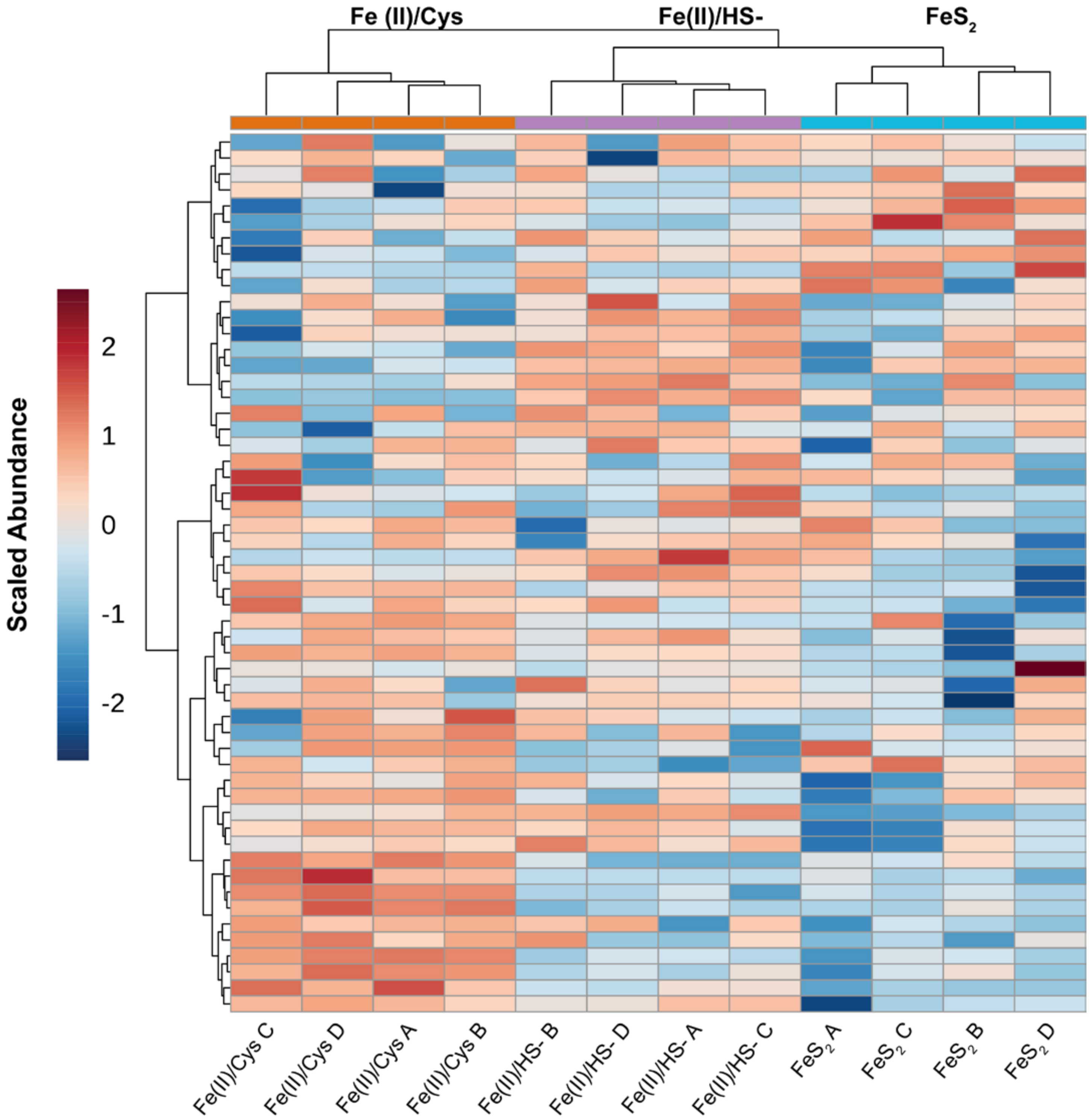

Figure S6. Hierarchical clustering heatmap of all iron-sulfur binding proteins. Dendrograms on each axis employ Euclidian distance and a ward agglomeration. Y axis dendrogram clusters by protein, while the x axis clusters by samples. Each block displays the relative (to the overall average) abundance in each sample (red> average, blue < average)
