# Supplementary material for: A shift between mineral and nonmineral sources of iron and sulfur causes proteome-wide changes in Methanosarcina barkeri
Source: Microbiol Spectr. 2024 Jan 5;12(2):e00418-23. doi: 10.1128/spectrum.00418-23 (PMC10846266; doi:10.1128/spectrum.00418-23)
Supplement: Figure S8 — Regulatory patterns of the mRNA transcripts and proteins of 3 key metal binding proteins. [file spectrum.00418-23-s0008.pdf]

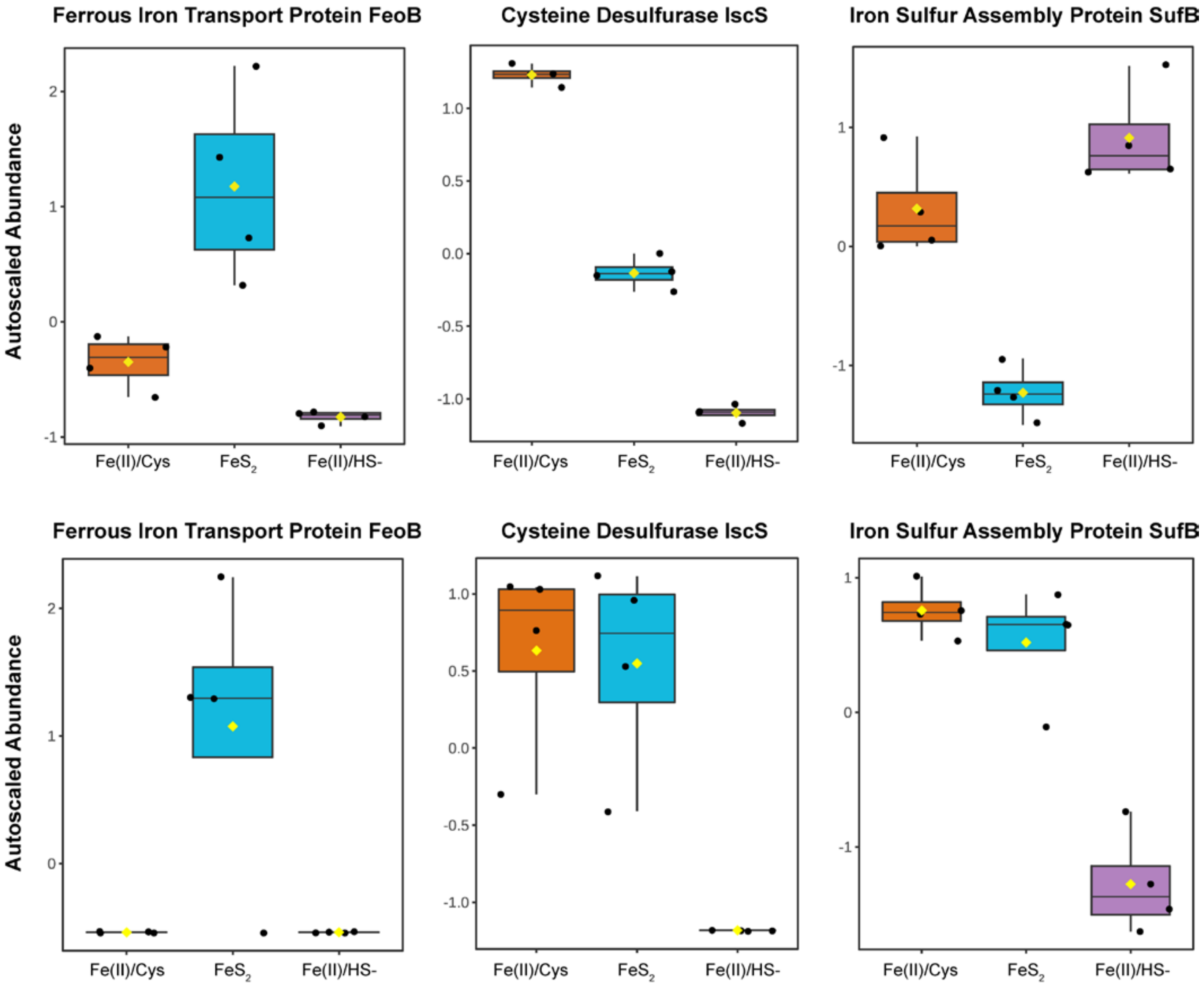

Figure S8. Regulatory patterns of the mRNA transcripts (top row) and proteins (bottom row) of 3 key metal binding proteins. Box and whisker plots show scaled abundance in each condition.
