# Supplementary material for: A shift between mineral and nonmineral sources of iron and sulfur causes proteome-wide changes in Methanosarcina barkeri
Source: Microbiol Spectr. 2024 Jan 5;12(2):e00418-23. doi: 10.1128/spectrum.00418-23 (PMC10846266; doi:10.1128/spectrum.00418-23)
Supplement: Tables S1 to S3 — Protein identifiers and abundance change of ribosomal proteins. [file spectrum.00418-23-s0009.docx]

**Table S1: Identifiers of proteins shown in Figure S1**

**Table S2. Ribosomal proteins showing significant abundance change in each pairwise comparison**

**Table S3. Identifier for top 30 Fe-S bindling proteins (filitered by p-value)**
